# Novel benzo-bis(1,2,5-thiadiazole) fluorophores for *in vivo* NIR-II imaging of cancer†
†Electronic supplementary information (ESI) available. See DOI: 10.1039/c6sc01561a


**DOI:** 10.1039/c6sc01561a

**Published:** 2016-06-16

**Authors:** Yao Sun, Chunrong Qu, Hao Chen, Maomao He, Chu Tang, Kangquan Shou, Suhyun Hong, Meng Yang, Yuxin Jiang, Bingbing Ding, Yuling Xiao, Lei Xing, Xuechuan Hong, Zhen Cheng

**Affiliations:** a State Key Laboratory of Virology , Key Laboratory of Combinatorial Biosynthesis and Drug Discovery (MOE) and Hubei Provincial Key Laboratory of Developmentally Originated Disease , Wuhan University School of Pharmaceutical Sciences , Wuhan 430071 , China . Email: xhy78@whu.edu.cn; b Molecular Imaging Program at Stanford (MIPS) , Bio-X Program, and Department of Radiology , Canary Center at Stanford for Cancer Early Detection , Stanford University , California 94305-5344 , USA . Email: zcheng@stanford.edu; c Chinese Academy of Medical Science , Peking Union Medical College Hospital , Department of Ultrasound , Beijing , 100730 , China

## Abstract

Optical imaging of diseases represents a highly dynamic and multidisciplinary research area, and second near-infrared window (NIR-II, 1000–1700 nm) imaging is at the forefront of the research on optical imaging techniques. Small-molecule based NIR-II (1000–1700 nm) dyes are highly promising candidates for *in vivo* molecular imaging because of their high biocompatibility, fast excretion, and high clinical translation ability. However, research reports on small-molecule based NIR-II dyes and probes are rare. Herein, we designed a series of fluorescent compounds (**Q1**, **Q2**, **Q3**, and **Q4**) and investigated the relationships between their structures and absorption/fluorescence properties. **Q4** (maximum emission at 1100 nm) stood out as the dye with the best physical properties and thus was selected as a scaffold for the facile construction of two types of water-soluble and biocompatible NIR-II probes (**Q4NPs** and **SCH1100**). Highly specific gastrin-releasing peptide receptor (GRPR) targeted NIR-II imaging of prostate cancer in living mice was achieved using the small-molecule probe **SCH1100**, which represents the first small peptide based NIR-II probe for targeted cancer imaging. The attractive imaging properties of **Q4**-based NIR-II probes open up many opportunities for molecular imaging and clinical translation in the unique NIR-II window.

## Abstract 2

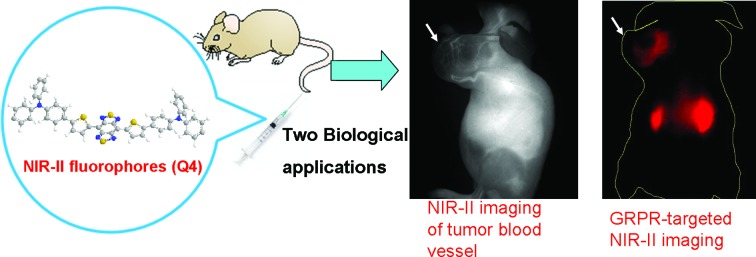
Optical imaging of diseases represents a highly dynamic and multidisciplinary research area, and second near-infrared window (NIR-II, 1000–1700 nm) imaging is at the forefront of the research on optical imaging techniques.

## Introduction

Optical imaging of diseases represents a highly dynamic and multidisciplinary research area. Over the last decade it has attracted extensive research attention from scientists working in a variety of fields such as chemistry, materials science, biotechnology, nanotechnology, biomedicine, *etc.*^1^–^3^ Optical imaging probes and techniques are expected to realize early cancer diagnosis and imaging guided therapy, and thus bring high impact to clinical cancer management.^4^,^5^ *In vivo* fluorescence imaging of biological systems in the second near-infrared window (NIR-II, 1000–1700 nm) is at the forefront of the research on optical imaging techniques, and it holds great promise owing to minimal autofluorescence and tissue scattering in this region, leading to deep tissue imaging capability, high spatial resolution, and high contrast.^6^,^7^ Moreover, recent studies suggest that fluorophores with emission in the NIR-II region can dramatically improve the imaging quality and signal-to-noise ratio compared to those used in the traditional NIR window I (NIR-I) region (650–900 nm).^8^,^9^ Developing novel NIR-II fluorophores and molecular probes for *in vivo* imaging applications thus has high significance and direct impact on the field of biomedicine.

To date, nanoparticle based systems, including single-walled carbon nanotubes (SWNTs),^10^,^11^ semiconducting quantum dots (QDs),^12^,^13^ rare-earth doped nanoparticles^14^ and conjugated polymers,^15^ have been actively explored for NIR-II fluorescence imaging. Considering the translation of NIR-II imaging agents into clinical applications, however, small-molecule based probes remain to be the most desirable and optimal candidates for this because of their high biocompatibility, fast excretion, quality control under the Current Good Manufacturing Practice (cGMP) conditions, and easy and robust preparation.^16^,^17^ Therefore, developing small-molecule based NIR-II fluorophores and probes with desirable chemical and physical properties, favorable excretion pharmacokinetics, minimal cellular toxicity, and clinical translation ability is crucial and highly demanded. It represents an emerging field in bioimaging and chemical research.

Developing small-molecule NIR-II dyes is conceptually straightforward but highly challenging in reality. After several years' efforts, we have recently reported a NIR-II fluorophore based on a small organic molecule (**CH1055**) that is rapidly excreted renally (90% excreted within 24 h).^18^ This compound was further successfully conjugated with a small protein, an anti-epidermal growth factor receptor (EGFR) affibody, for molecular imaging of abnormalities *in vivo*. The resulting probe achieved superior tumor imaging quality and tumor-to-background signal ratios. However, the capability of using **CH1055** as a generic NIR-II reporter for broad use remains unknown, especially using **CH1055** for the modification of small peptides or small molecules has not been demonstrated. Moreover, despite this promising example, other small-molecule NIR-II agents for *in vivo* imaging have not been reported.

**Fig. 1:**
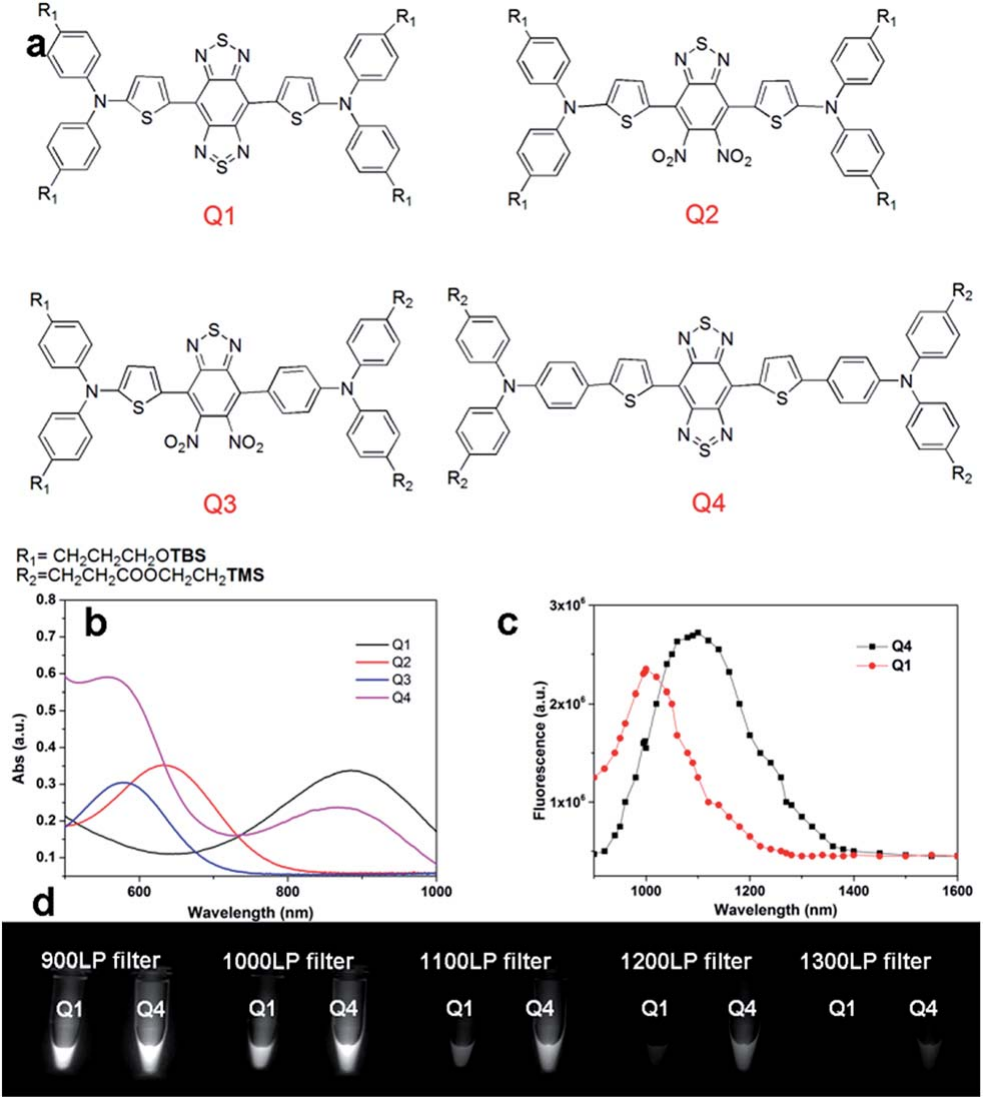
(a) Design of NIR-II dyes based on the D–A–D scaffold and the chemical structures of **Q1–Q4**, TBS = Si(Me)_2_^*t*^Bu, TMS = SiMe_3_; (b) UV absorbance of **Q1–Q4**; (c) NIR-II fluorescence emission of **Q1** and **Q4** with peaks at ∼1000 nm and ∼1100 nm under 808 nm excitation (exposure time: 10 ms); the emission of **Q2** and **Q3** is in the NIR-I region (Fig. S2,† data not shown here). (d) NIR-II signals of **Q1** and **Q4** with various long-pass (LP) filters (900–1400 nm).

Hence, in this work, we design a new type of NIR-II dye with a different core structure from **CH1055**. The donor–acceptor–donor (D–A–D) type core is the key for obtaining NIR-II dyes.^19^ Based on the D–A–D scaffold, herein we incorporated a thiophene spacer to design and synthesize a small library of fluorescent compounds (**Q1**, **Q2**, **Q3**, and **Q4**) and investigated the relationships between their structures and absorption/fluorescence properties (Fig. 1a). Furthermore, the most promising dye, **Q4**, of these four compounds was identified and used to prepare two distinctive NIR-II probes. One probe includes **Q4** encapsulated organic nanoparticles (**Q4NPs**) for NIR-II imaging of tumor blood vessels, the other is composed of **Q4** successfully conjugated with a small bombesin peptide to prepare the probe **SCH1100** for gastrin-releasing peptide receptors (GRPR) targeted NIR-II imaging of prostate cancer in living mice. These novel small-molecule based NIR-II dyes expand the library of NIR-II fluorophores to meet the demands of using the NIR-II imaging technique for a variety of applications.

## Results and discussion

We first incorporated an electron-rich thiophene spacer relative to benzene in all four compounds (**Q1–Q4**), because a thiophene moiety facilitates an intramolecular charge transfer (ICT), resulting in a further bathochromic shift.^20^ The synthetic route of **Q1–Q4** was more than 10 steps (see ESI†). Key steps utilized to assemble the core structure of the targeted compounds included Stille coupling, a Suzuki coupling reaction, iron reduction and *N*-thionylaniline induced ring closure. All compounds were verified by NMR and ESI-MS, and also exhibited good solubility in common organic solvents such as CH_2_Cl_2_ and THF. Functional groups such as carboxylic acid groups were introduced into these compounds to impart a certain aqueous solubility and allow facile conjugation to biomolecules, which expanded their *in vivo* imaging applications (see ESI†). The HOMO, LUMO, and band gap levels of **Q1–Q4** analogs (to reduce the computational cost, R_1_ and R_2_ groups were replaced by a simple methyl group) were obtained from theoretical calculations (Tables 1 and S1†). The LUMOs of all compounds showed a strong contribution at the electron accepting aromatic moieties (benzo-bis(1,2,5-thiadiazole)). The HOMOs are well delocalized along the whole backbone for **Q1** and **Q4**. Moreover, **Q1** and **Q4** presented higher-lying calculated HOMO and LUMO levels and showed lower band gaps compared to those of **CH1055**, as the electron-rich thiophene spacer significantly lowers the oxidation potential, accounting for the relatively stronger ICT effect.

**Table 1:** Comparison of HOMO and LUMO orbital surfaces of **CH1055**, **Q1**, **Q2**, **Q3** and **Q4** using DFT B3LYP/6-31G(d) scrf = (cpcm, solvent = dichloromethane) method. To reduce the computational cost, R substituent groups were replaced by methyl, *E*_gap_ = *E*_LUMO_ – *E*_HOMO_

Compound	HOMO	Energy (eV)	LUMO	Energy (eV)	*E*_gap_ (eV)
**CH1055** (R = CH_3_)	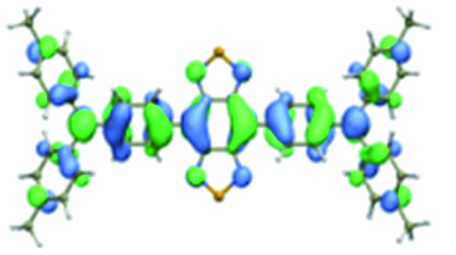	–4.75	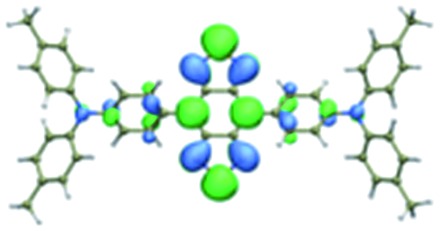	–3.26	1.49
**Q1** (R = CH_3_)	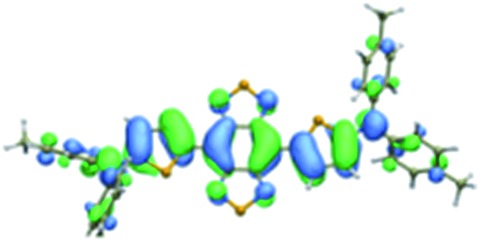	–4.37	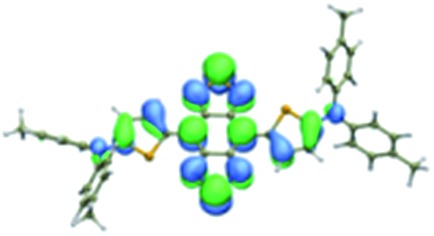	–3.28	1.09
**Q2** (R = CH_3_)	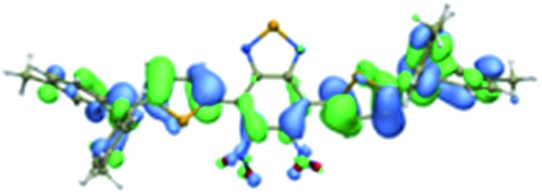	–4.97	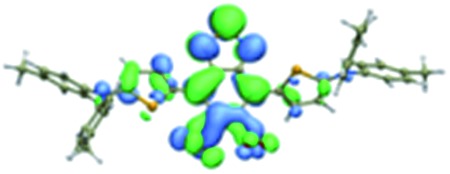	–3.07	1.90
**Q3** (R = CH_3_)	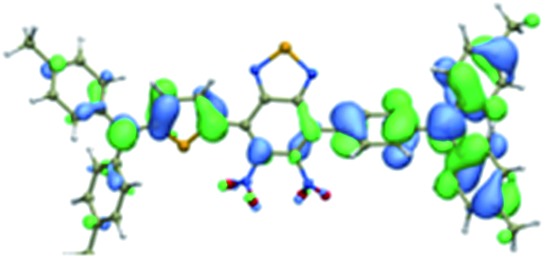	–5.03	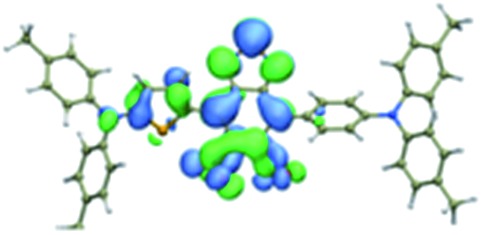	–3.06	1.97
**Q4** (R = CH_3_)	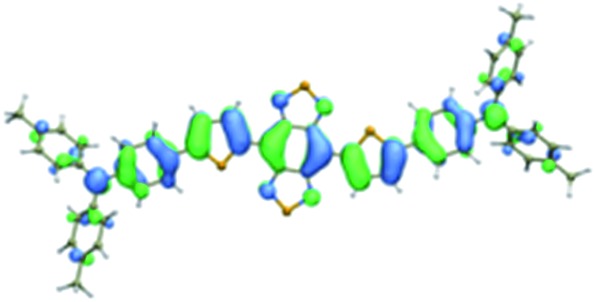	–4.58	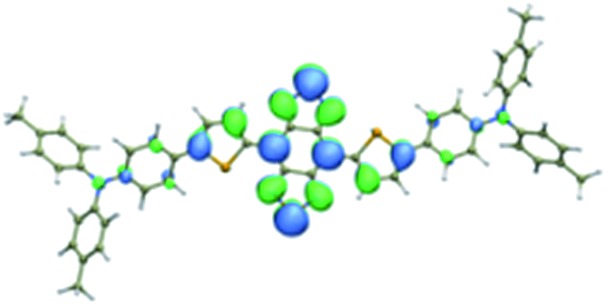	–3.46	1.12

The UV-vis-NIR absorption spectrum of **Q1–Q4** was measured in DCM. The absorption bands of **Q1** and **Q4** were at 800–1000 nm because of the formation of a strong charge-transfer structure between their D–A–D units (Fig. 1b). Meanwhile, NIR-II emission signals were only observed in **Q1** and **Q4** solutions under 808 nm excitation and with a 1000 long-pass (LP) filter (Fig. S1†), whereas **Q2** and **Q3** displayed NIR-I emission (Fig. S2†) and were not suitable for further NIR-II imaging applications. The fluorescence emission spectra of **Q4** and **Q1** were measured and they demonstrated a peak emission wavelength at ∼1100 nm and ∼1000 nm, respectively (Fig. 1c). Finally, we compared the NIR-II fluorescence signals of **Q1** and **Q4** under various LP filters (900–1400 nm, Fig. 1d). The results indicated that the fluorescence signals of **Q4** were stronger than those of **Q1** with each filter, and no signals were observed for either compound with a 1400 nm filter. All these data have demonstrated that **Q4** is a new type of promising NIR-II fluorescence compound, which is suitable for further imaging applications.

**Fig. 2:**
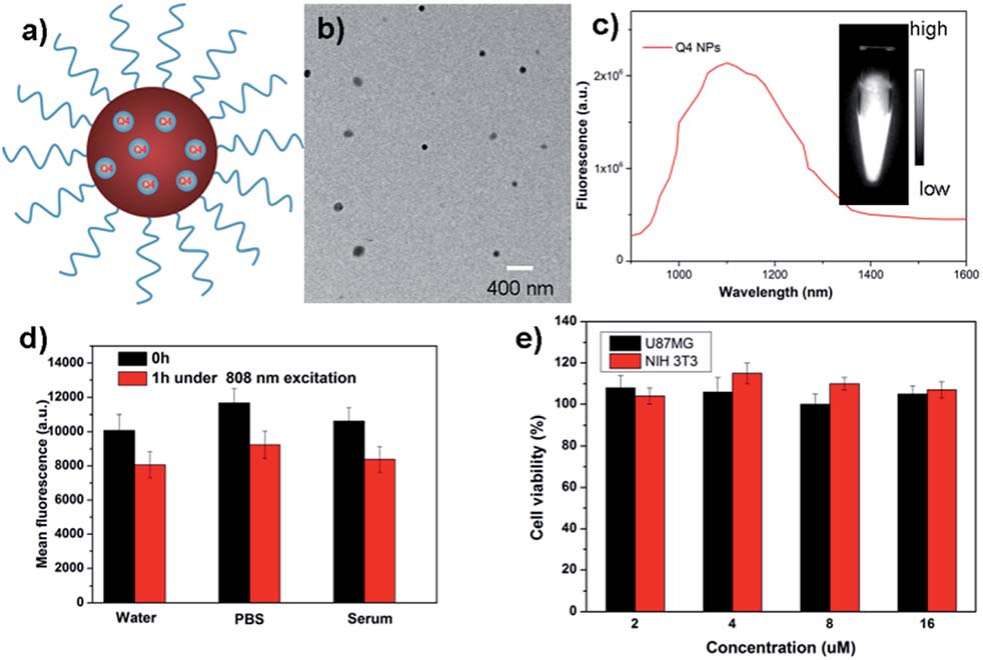
(a) A schematic design of **Q4NPs** showing **Q4** molecules loaded in the DSPE-mPEG NPs. (b) The TEM image of the **Q4NPs**. (c) Fluorescence emission of **Q4NPs** under 808 nm excitation. (d) Photostability of **Q4NPs** in different media including water, PBS and serum under continuous 808 nm excitation for 1 h. (e) Cellular toxicity of **Q4NPs** in U87MG and NIH-3T3 cell lines.

**Q4** was then encapsulated into a PEGylated surfactant DSPE-mPEG5000 to prepare organic nanoparticle based water-soluble and biocompatible NIR-II nanoprobes, **Q4NPs** (Fig. 2a, see ESI†). The synthesized **Q4NPs** showed high monodispersity and homogeneity with an average particle size of ∼60.0 nm as determined by transmission electron microscopy (TEM, Fig. 2b) and a hydrodynamic diameter of ∼70.0 nm as determined by dynamic light scattering (DLS, Fig. S3†). The fluorescence emission spectrum of **Q4NPs** demonstrated a similar emission wavelength at ∼1100 nm as **Q4** (Fig. 2c). The **Q4NPs** also exhibited high photostability in phosphate-buffered saline (PBS), water and mouse serum with negligible decay under continuous excitation for 1 h (Fig. 2d). The prepared **Q4NPs** were highly stable and can be stored in PBS without any precipitation in the refrigerator for one month. The result of the cytotoxicity study further indicated the high viability of U87MG and NIH-3T3 cells after 48 h of incubation with **Q4NPs** (2, 4, 8 and 16 μM), demonstrating the high biocompatibility of **Q4NPs** (Fig. S4†).

**Fig. 3:**
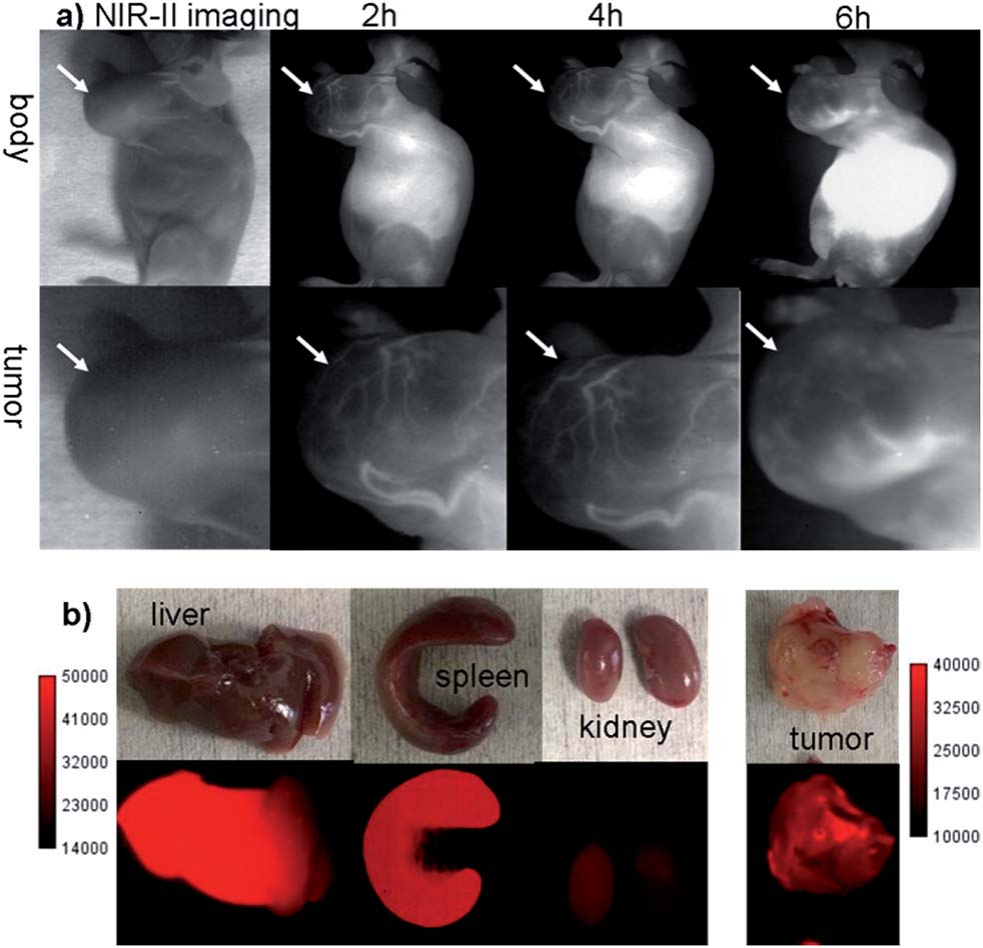
(a) The NIR-II images of the blood vessels of the U87MG tumour (*n* = 3) at different time points after a tail vein injection of **Q4NPs** under 808 nm excitation (1000 LP and 100 ms); white arrows indicate the tumor. (b) The ex-biodistribution of **Q4NPs** in the liver, spleen, kidney and tumor after 96 h under 808 nm excitation (1000 LP and 200 ms).

The U87MG tumor-bearing nude mice (*n* = 3) were injected with 100 μg of **Q4NPs** (0.0013 nmol, containing 24 μg of the **Q4** molecule, calculated based on the UV-Vis measurement of **Q4**). Interestingly, the blood vessels of the tumor could be clearly visualized from the surrounding background tissue at 2 h p.i. using NIR-II imaging (Fig. 3a and S5†). After 6 h, the fluorescence signal and image quality of the tumor blood vessels reduced significantly, whereas a higher signal intensity was observed inside the tumor because of the non-specific diffusion and accumulation of **Q4NPs** in the tumor (Fig. S6†). This phenomenon could be explained by the passive targeting mechanism and enhanced permeability and retention (EPR) effect. The promising imaging result of the NIR-II **Q4NP** probe highlighted its possible use for monitoring tumor vasculatures and the EPR effect, which has not been achieved by previous NIR-I and NIR-II imaging in the literature (Fig. 3a, S5 and S6†). *Ex vivo* biodistribution studies were further performed at 96 h post-injection of the nanoprobe to evaluate the distribution of **Q4NPs** in major organs (Fig. 3b and S7†). It was found that **Q4NPs** mainly accumulated in the liver and spleen, suggesting the clearance routes of **Q4NPs** are predominantly through hepatobiliary systems, which is consistent with the clearance routes of many nanoparticle-based probes.^21^ In addition, a similar level of accumulation was observed in the tumor, indicating that **Q4NPs** can passively target tumors and be used for cancer theranostic applications (Fig. 3 and S7†).

**Fig. 4:**
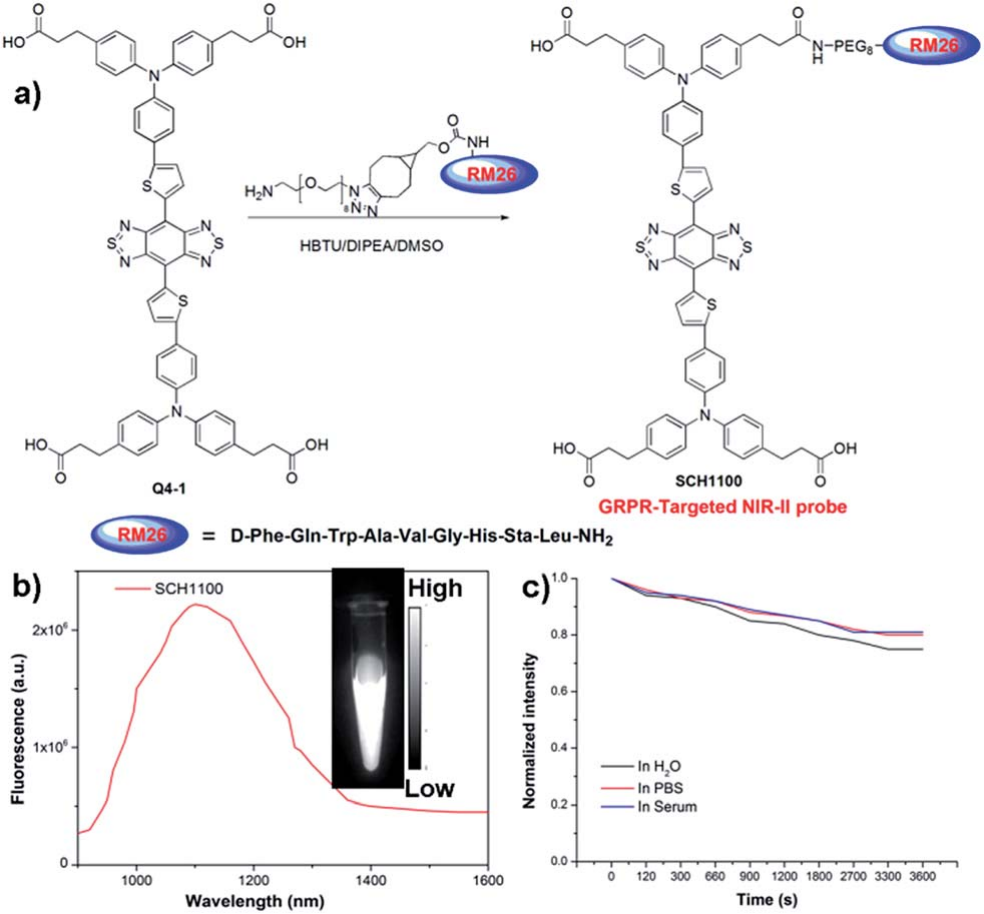
(a) Conjugation of **Q4-1** with NH_2_-PEG8-RM26 peptide to prepare a GRPR targeted probe, **SCH1100**; (b) fluorescence emission of **SCH1100** under 808 nm excitation; (c) photostability curves of **SCH1100** in water, PBS and serum under 808 nm laser illumination for 1 h.

We next demonstrated the application of **Q4** for receptor-targeted imaging of tumors such as prostate cancer (PCa). PCa is one of the leading causes of cancer-related death among men in the United States and Europe.^22^ If diagnosed early, chances for survival increase tremendously.^23^ In addition, imaging-guided fluorescence surgery would also be helpful to reduce the re-occurrence of PCa.^24^ GRPR has been reported to be overexpressed in PCa, and can serve as a promising target for PCa theranostics.^25^,^26^ Considering the advantages of NIR-II imaging, we explored the development of a novel small-molecule based GRPR-targeted NIR-II probe **SCH1100**, and investigated its imaging properties *in vivo*. **SCH1100** was prepared through the direct conjugation of one of the carboxylic acid groups of **Q4-1** (see ESI†) with a GRPR targeting ligand RM26 peptide. RM26 has been demonstrated as a promising targeting peptide for GRPR by our group and other groups.^27^,^28^ In order to increase the solubility of the **SCH1100** probe, cyclooctyne functionalized RM26 was firstly conjugated with a small NH_2_-PEG_8_-azide through a Cu-free click reaction, and then amidation with one of the carboxylic acid groups of Q4-1 was performed to obtain **SCH1100** (Fig. 4a and ESI†). **SCH1100** was purified using HPLC and characterized using MALDI-TOF-MS [calcd. for C_146_H_176_N_24_O_28_S_4_: 2843.3630, found: *m*/*z* 2844.1526 (Fig. S8†)]. The fluorescence emission spectrum of **SCH1100** demonstrated the maximum emission wavelength at ∼1100 nm (Fig. 4b). The fluorescence quantum yield of **SCH1100** in water solution under an excitation of 808 nm was ∼0.2%, measured against a standard IR-26 dye as a reference (Fig. S9†). **SCH1100** also exhibited high photostability in PBS, water and mouse serum with a slight decay under continuous excitation for 1 h (Fig. 4c). Furthermore, the high viability of human PCa cell lines (PC3) and NIH-3T3 cells after 24 h incubation with different concentrations of **SCH1100** demonstrated the high biocompatibility of **SCH1100***in vitro* (Fig. S10†). These results indicated that **SCH1100** as an aqueous soluble, photo-stable and biocompatible NIR-II fluorescence probe is suitable for biological imaging.

**Fig. 5:**
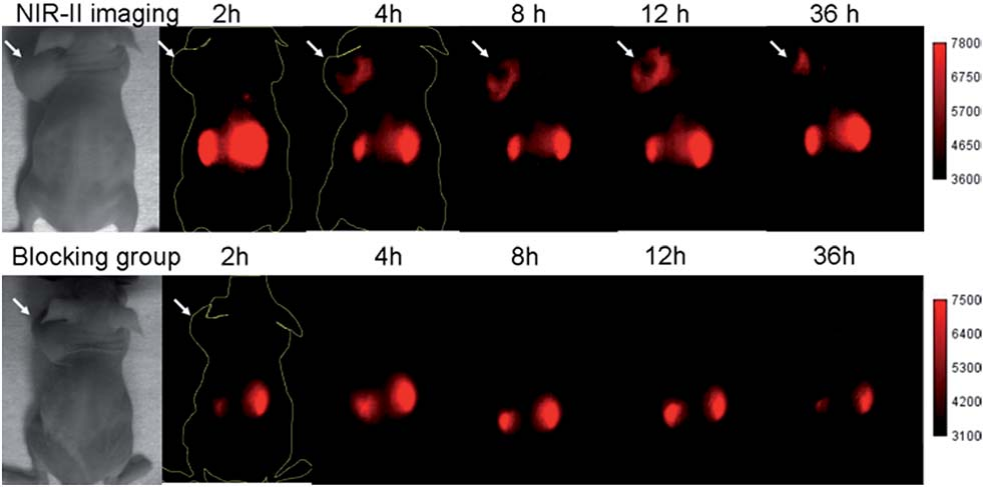
The NIR-II images of PC3 tumor mice (*n* = 3) at different time points (2, 4, 8, 12, 36 h) after tail vein injection of **SCH1100** with or without blocking agent RM26 (400 μg) under 808 nm excitation (1000 LP and 500 ms); white arrows indicate the tumor.

**SCH1100** was then intravenously injected (100 μg) in PC3 tumor-bearing mice (*n* = 3 per group). From NIR-II imaging, the subcutaneous PC3 tumor could be clearly visualized from the surrounding background tissue from 4–36 h p.i. (Fig. 5, 1000 LP, 500 ms), and the tumor uptake reached maximum at 12 h. The targeting specificity of **SCH1100** for GRPR was confirmed by the blocking experiment, and the tumor fluorescence signals were successfully reduced at all time points after co-injection of RM26 peptide (400 μg) with **SCH1100** for NIR-II imaging (Fig. 5). An *ex vivo* biodistribution study was performed for **SCH1100** to evaluate its distribution in major organs at 48 h (Fig. S11†). High accumulation was observed in the liver and kidney (ROI analysis indicated the fluorescence signal ratio in kidney/liver is ∼2), which suggested that the clearance routes of **SCH1100** were through both hepatobiliary and renal systems, which was different from **Q4NPs**. In addition, the uptake of **SCH1100** in the tumor was far higher than in most of normal organs except the kidneys, which further confirmed the excellent GRPR-targeting ability and specificity of **SCH1100**. Hence, **SCH1100** represents a highly promising and clinically translatable NIR-II fluorescence probe for inexpensive and rapid detection and monitoring of PCa.

## Conclusions

In conclusion, we have successfully developed a novel and versatile small-molecule based NIR-II fluorophore **Q4**, which shows to be highly promising for molecular imaging and clinical translation. Using this scaffold, organic nanoparticle based NIR-II imaging probes **Q4NPs** have been prepared, which allowed for *in vivo* and high resolution imaging of the blood vessels of the tumor, which has not been achieved in the NIR-I and NIR-II window before. Furthermore, a novel GRPR-targeted small NIR-II imaging probe **SCH1100** has been successfully prepared and demonstrated specific GRPR-targeted imaging of PCa *in vivo*. The novel organic fluorescent compound **Q4** provides unprecedented opportunities for constructing a variety of NIR-II probes for *in vivo* molecular imaging.

## Supplementary Material

Supplementary information
