# Supplementary material for: Novel benzo-bis(1,2,5-thiadiazole) fluorophores for in vivo NIR-II imaging of cancer
Source: Chem Sci. 2016 Jun 16;7(9):6203–7. doi: 10.1039/c6sc01561a (PMC6024204; doi:10.1039/c6sc01561a)
Supplement: Supplementary file 1 [file SC-007-C6SC01561A-s001.pdf]

Electronic Supplementary Material (ESI) for Chemical Science.
This journal is © The Royal Society of Chemistry 2016

Supporting Information

Novel benzo-bis(1,2,5-thiadiazole) fluorophores for *in vivo* NIR-II imaging of cancer

Yao Sun[1,2†], Chunrong Qu[1†], Hao Chen[2], Maomao He[1], Chu Tang[2], Kangquan Shou[2], Suhyun Hong[2], Meng Yang[3], Yuxin Jiang[3], Bingbing Ding[1], Yuling Xiao[1], Lei Xing[2], Xuechuan Hong[*1], and Zhen Cheng[*2]

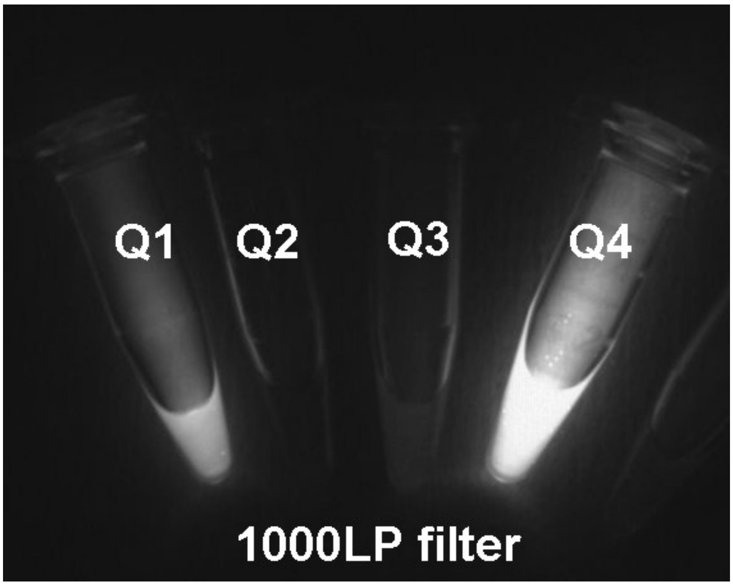

Figure S1. Fluorescent signals of Q1 to Q4 were performed with an 808 nm excitation and an 1000 nm long-pass (LP) filter (Thorlab).

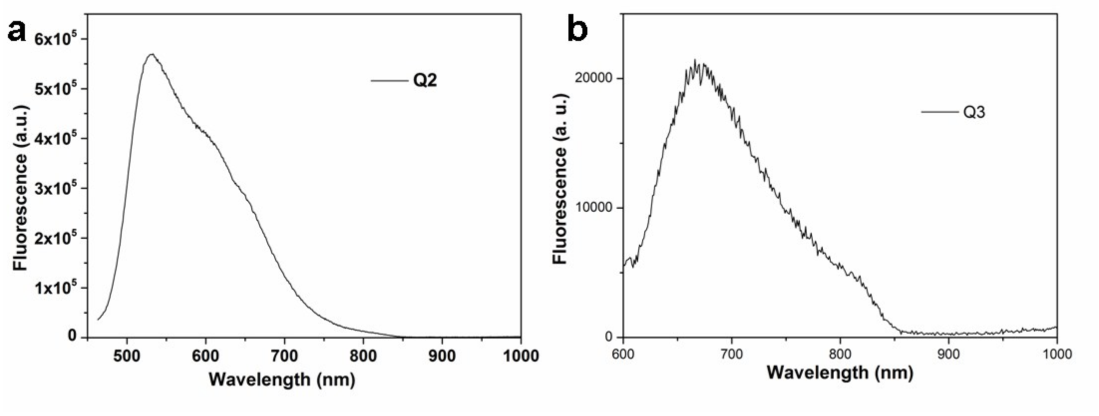

Figure S2. Emission spectra of Q2 to Q3.

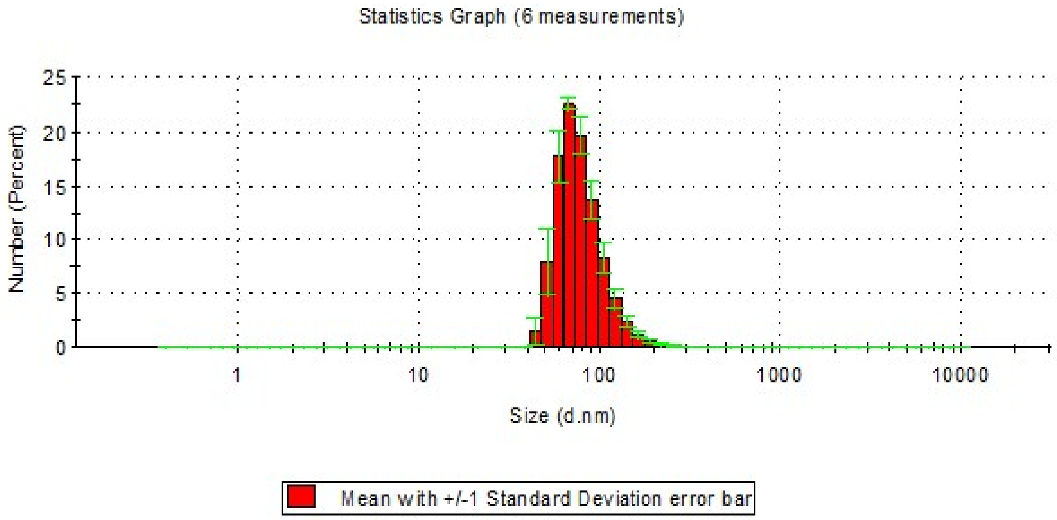

Figure S3. The size distribution of **Q4NPs** in water based on DLS measurement (repeated 6 times)

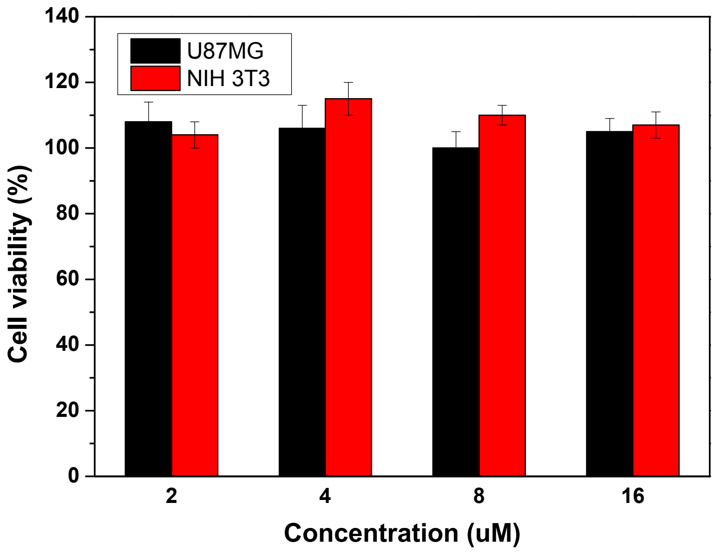

Figure S4. Cellular toxicity of **Q4NPs**. Cell toxicity was assayed utilizing the U87MG and NIH 3T3 cell lines.

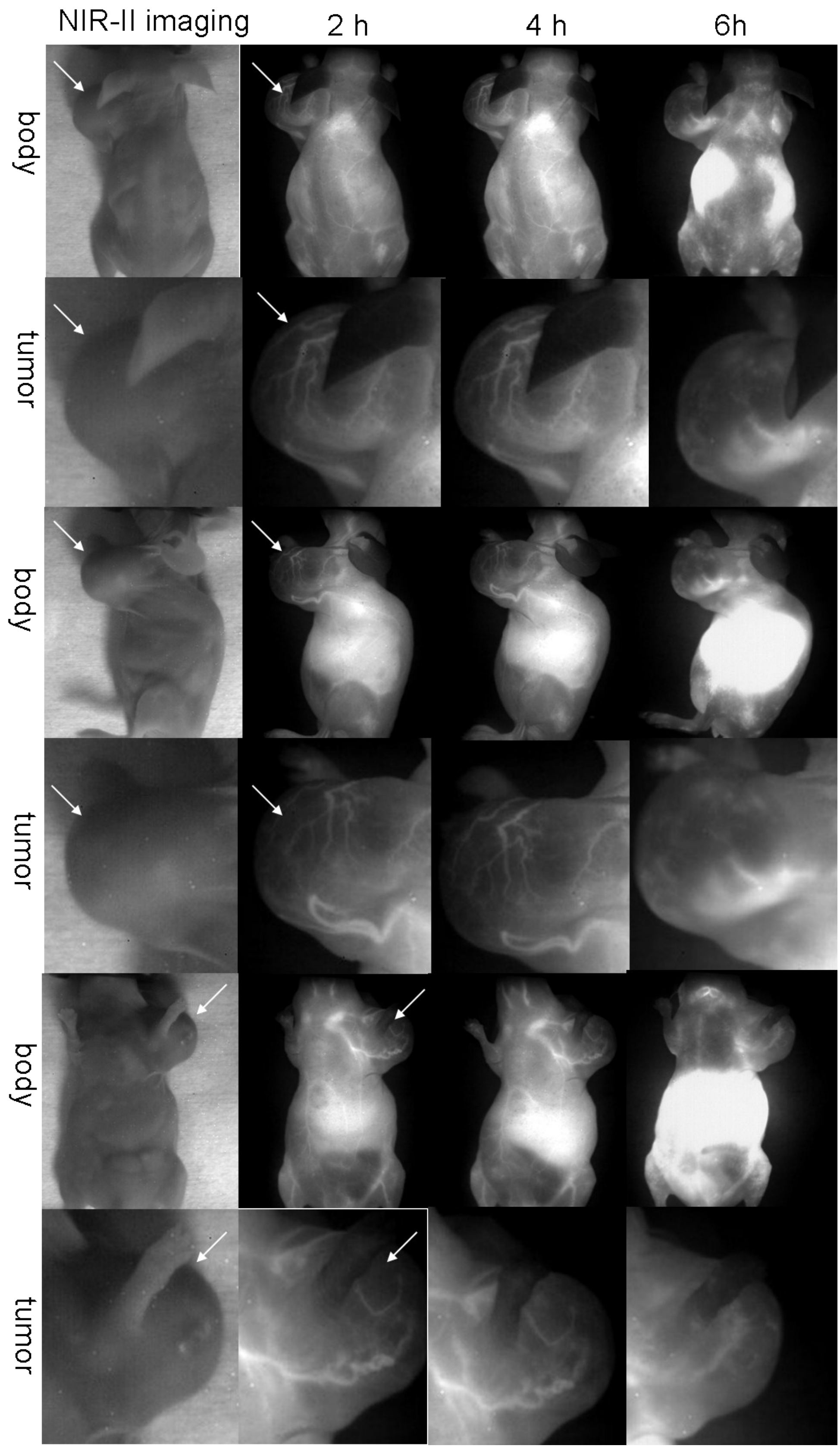

Figure S5. The NIR-II images of blood vessel of U87MG tumors at different time point after tail vein injection of **Q4NPs** under an 808 nm excitation (1000LP and 100 ms), white arrows indicate the tumor.

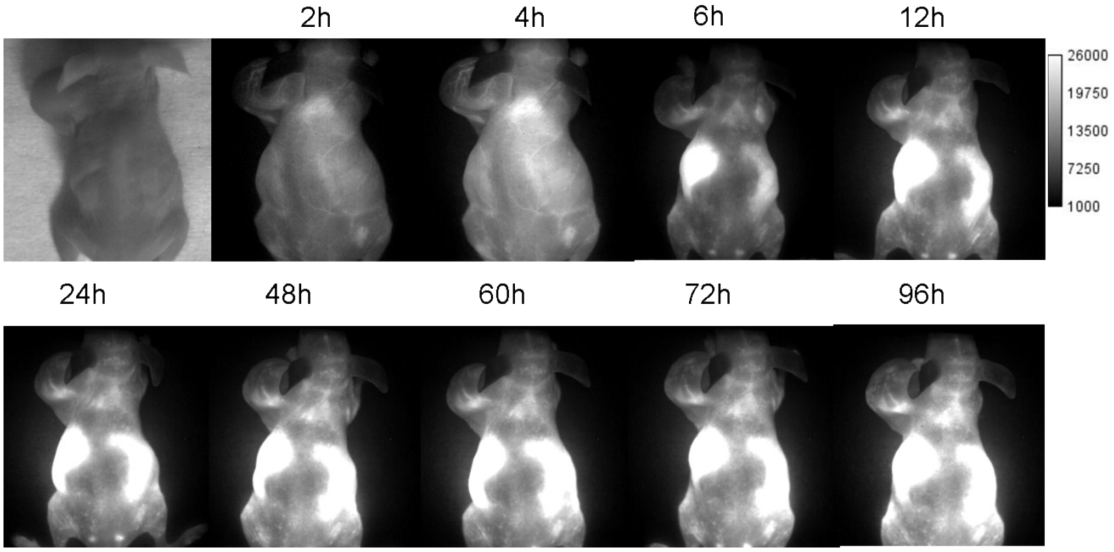

Figure S6. The NIR-II images of U87MG tumors at different time points after tail vein injection of **Q4NPs** under an 808 nm excitation (1000LP and 100 ms).

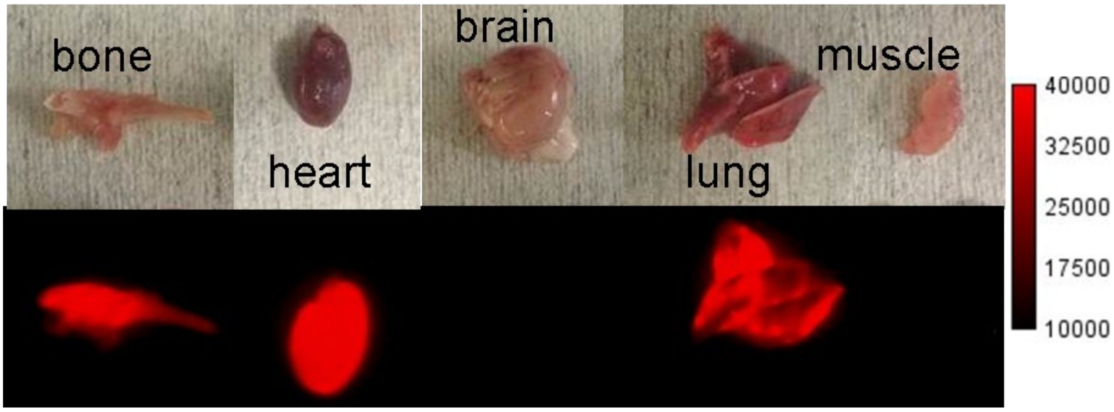

Figure S7. The ex-vivo biodistribution of **Q4NPs** in tumor mice at 96 h under an 808 nm excitation (1000LP and 200 ms).

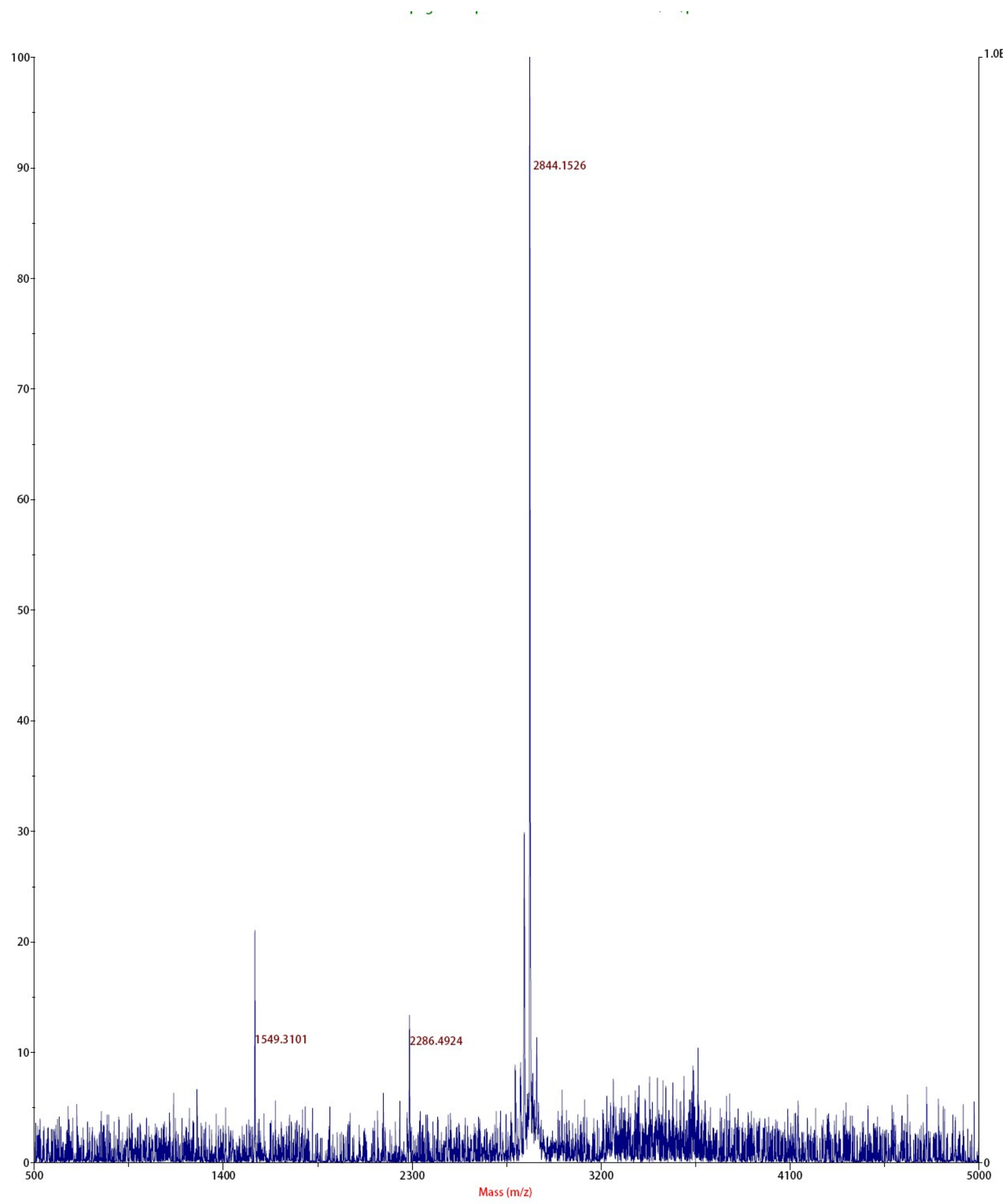

Figure S8. MALDI-TOF-MS for **SCH1100**

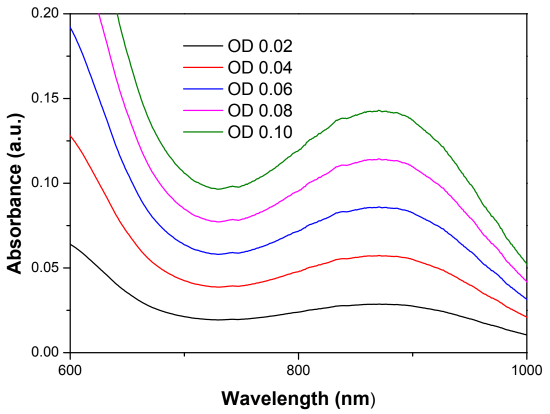

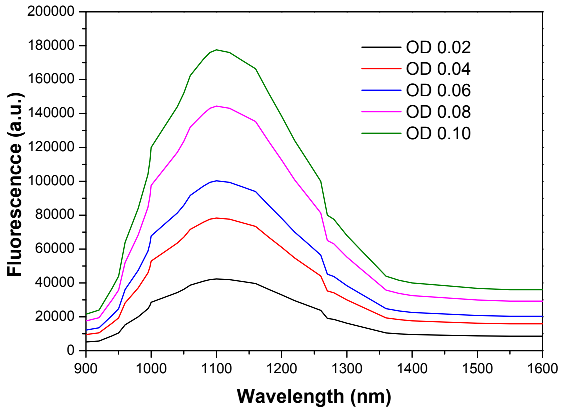

Figure S9. Quantum yield Measurements of SCH1100. In order to measure the quantum yield of SCH1100, a reference IR-26 (0.5%) was chosen.[1] Five difference concentrations at or below OD 0.1 (roughly OD 0.1, 0.08, 0.06, 0.04, and 0.02) were measured and the integrated fluorescence was plotted against absorbance for both IR-26 and SCH1100. Comparison of the slopes led to the determination of the quantum yield of SCH1100. The quantum yield was calculated in the following manner:

$$QY = QY_{ref} \times \frac{n^2}{n_{ref}^2}\left(\frac{A_{ref}}{I_{ref}}\right)\frac{I_{sample}}{A_{sample}}$$

Reference: A. L. Antaris, H. Chen, K. Cheng, Y. Sun, G. S. Hong, C. R. Qu, S. Diao, Z. X. Deng, X. M. Hu, B. Zhang, X. D. Zhang, O. K. Yaghi, Z. R. Alamparambil, X. C. Hong, Z. Cheng and H. J. Dai, *Nat. Mater.,* 2016, **15**, 235-242.

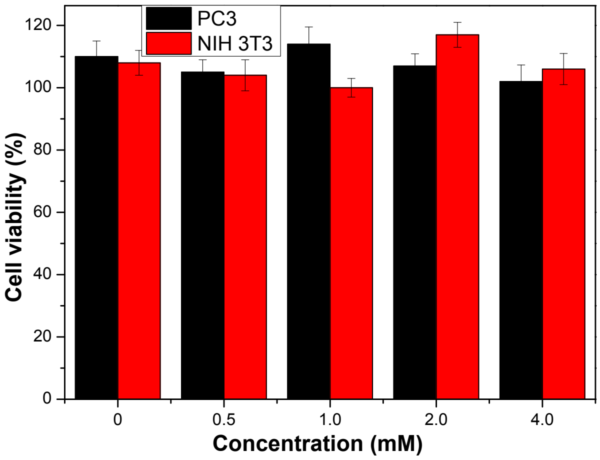

Figure S10. Cellular toxicity of **SCH1100**. Cell toxicity was assayed utilizing the PC3 and NIH 3T3 cell lines.

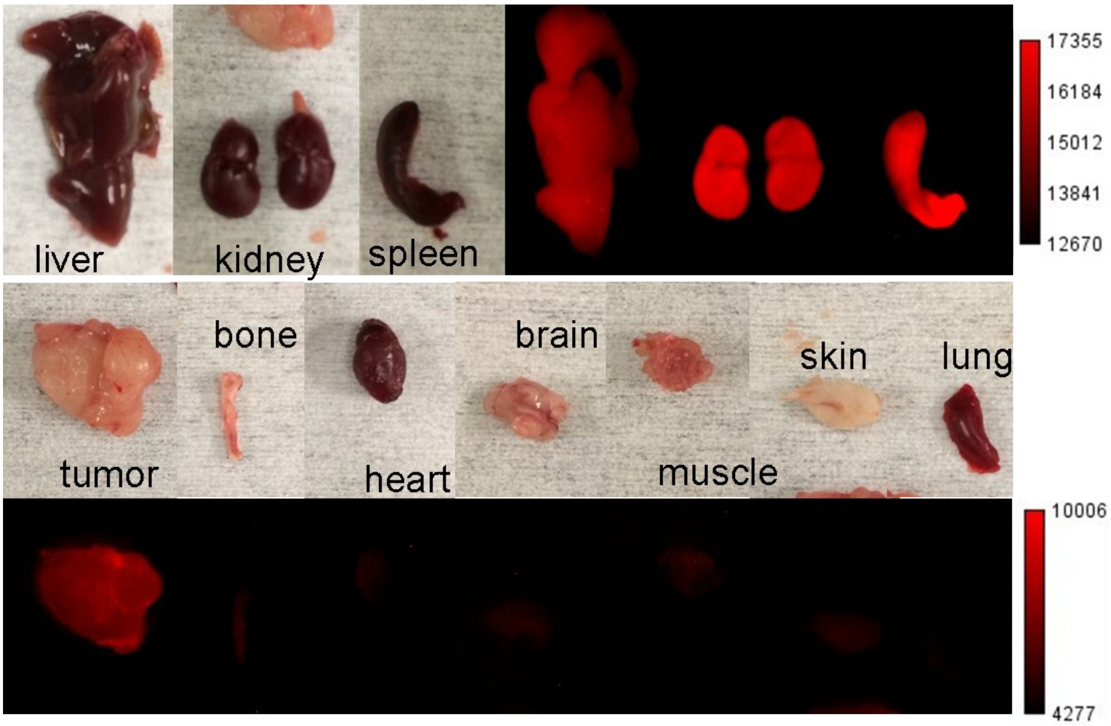

Figure S11. The biodistribution of **SCH1100** in tumor mice at 48 h under an 808 nm excitation (1000LP and 800 ms).

Compound	HOMO	Energy (eV)	LUMO	Energy (eV)	E_{gap} (eV)
$CH1055\text{-}CH_3$ 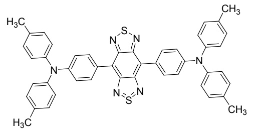	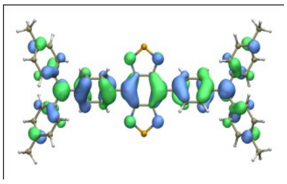	-4.75	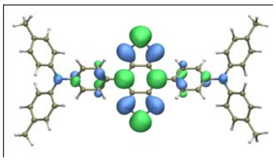	-3.26	1.49
$Q1\text{-}CH_3$ 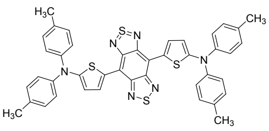	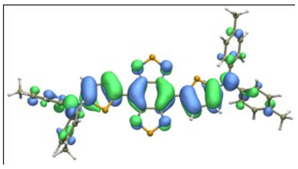	-4.37	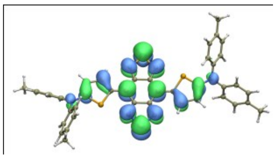	-3.28	1.09
$Q2\text{-}CH_3$ 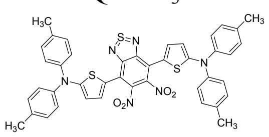	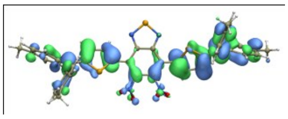	-4.97	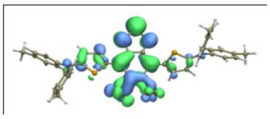	-3.07	1.90
$Q3\text{-}CH_3$ 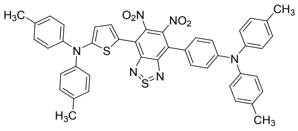	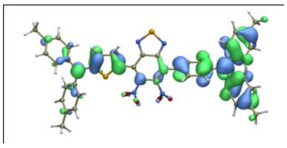	-5.03	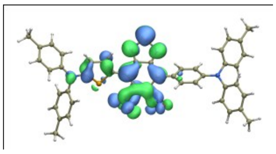	-3.06	1.97
$Q4\text{-}CH_3$ 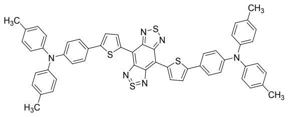	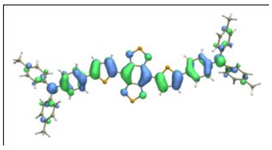	-4.58	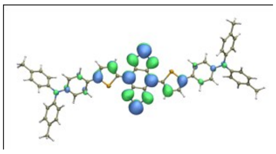	-3.46	1.12
Q4 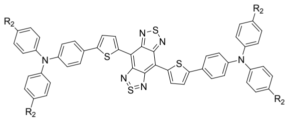 R_2=$CH_2CH_2COOCH_2CH_2TMS$	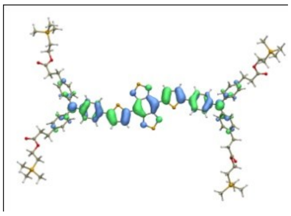	-4.62	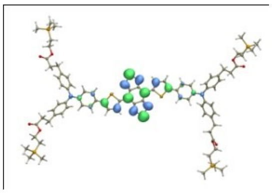	-3.48	1.14

Table S1. Comparison of HOMO and LUMO orbital surfaces of CH1055, Q1, Q2, Q3 and Q4 and Q4 without structural simplification using DFT B3LYP/6-31G(d) scrf=(cpcm, solvent=dichloromethane) method. To reduce the computational cost, R1 and R2 substituent groups were replaced by methyl. $E_{gap}=E_{LUMO}-E_{HOMO}$

General methods

All chemicals were purchased from commercial sources (such as Aldrich, conju-probe and Lumiprobe). The ^{1}H and ^{13}C NMR spectra were acquired on a Bruker 400 MHz magnetic resonance spectrometer. Data for ^{1}H NMR spectra are reported as follows: chemical shifts are reported as δ in units of parts per million (ppm) relative to chloroform-d (δ 7.26, s); multiplicities are reported as follows: s (singlet), d (doublet), t (triplet), q (quartet), dd (doublet of doublets), m (multiplet), or br (broadened); coupling constants are reported as a J value in Hertz (Hz); the number of protons (n) for a given resonance is indicated nH, and based on the spectral integration values. MALDI-MS spectrometric analyses were performed at the Mass Spectrometry Facility of Stanford University. HPLC was performed on a Dionex HPLC System (Dionex Corporation) equipped with a GP50 gradient pump and an in-line diode array UV-Vis detector. A reversed-phase C18 (Phenomenax, 5 μm, 4.6 × 250 mm, 5 μm, 10 × 250 mm or 21.2 × 250 mm) column was used for analysis and semi-preparation. UV absorbance of the probe was recorded on an Agilent 8453 UV spectrophotometer. Fluorescence was recorded on a Fluoromax-3 spectrafluorometer (Jobin Yvon). Transmission electron microscopy (TEM) images were recorded on a JEOL 2010 transmission electron microscope at an accelerating voltage of 100 kV. The TEM specimens were made by placing a drop of the nanoparticle aqueous solution on a carbon-coated copper grid. The hydrodynamic size was determined by dynamic light scattering (DLS) using a 90 plus particle size analyzer (Malvern, Zetasizer Nano ZS90).

Calculation of the number of Q4 molecule in one Q4NPs. The number of Q4 molecule in one Q4NPs was calculated as follows. Considering the length of PEG (MW=5000) was 11.2 nm (reference: L. Sportelli, *Biochim. Biophys. Acta.* **2003**, 1615, 33-59) and the average size of Q4NPs was ~60.0 nm, the diameter of the Q4 core in Q4NPs was about 38 nm. Based on the density of Q4 (1.1 g/cm^3), the number of Q4 molecule in one Q4NPs can be finally calculated to be about 1.2×10^4. The equation was listed as follows:

$$N_{Q4} = \frac{W_{Q4core} \bullet N_A}{M_{Q4}} = \frac{V_{Q4core} \bullet \rho_{Q4} \bullet N_A}{M_{Q4}} = \frac{(4/3)\pi r^3 \bullet \rho_{Q4} \bullet N_A}{M_{Q4}}$$

In this equation, N_{Q4} is the number of Q4 molecule in one Q4NPs, W_{Q4core} is the weight of the Q4 core in one Q4NPs, V_{Q4} is the volume of the Q4 core in one Q4NPs, M_{Q4} is the molar mass of Q4 (1533 g/mole), r is the radius of Q4core (19 nm), ρ is the density of Q4 (1.1 g/cm^3), NA is Avogadros constant (6.02×10^{23}).

Formula reference: Fan, Q. et. al. *Advanced. Materials,* 2015, 27(5):843-847.

PL excitation spectra. PLE spectrum of the Q1, Q4, Q4NPs and SCH1100 solution was taken using a home-built NIR-II spectroscopy setup.

Cell line and animal model. U87MG glioblastoma cells, PC-3 cells and NIH-3T3 cells were obtained from the American Type Culture Collection (Manassas, VA, USA) and culture media was obtained from Invitrogen Co. (Carlsbad, CA, USA). The cells were cultured in Dulbecco's modified Eagle's medium (DMEM) supplemented with 10% (v/v) fetal bovine serum and 1% (v/v) penicillin at 37°C and 5% CO_2. The U87MG or PC-3tumor model were established by subcutaneous injection of U87MG cells or PC-3 cells (~5×10^6 in 100 μL of PBS) into the front flank of female or male athymic nude mice (Harlan). The mice were subjected to imaging studies when the tumor volume reached 200-500 mm^3 (about 4 weeks after inoculation). Animal experiments were performed according to a protocol approved by the Stanford University Institutional Animal Care and Use Committee.

Cell viability. *In vitro* cytotoxicity of Q4NPs or SCH1100 was determined in U87MG or PC-3 or NIH-3T3 cells by the MTT assay. U87MG or PC-3 or NIH-3T3 cells were incubated on 96-well plate in DMEM medium containing 10% FBS and 1% penicillin/streptomycin at 37 °C in 5% CO_2 humidified atmosphere for 24 h and 0.5×10^4 cells were seeded per well. Cells were then cultured in the medium supplemented with indicated doses of different Q4NPs or SCH1100 for 24 h. The final concentrations of Q4NPs in the culture medium were fixed at 2, 4, 8 and 16 μM in the experiment. The final concentrations of SH1100 in the culture medium were fixed at 0.5, 1.0, 2.0 and 4.0 mM in the experiment. Addition of 10 μL of MTT (0.5 mg/mL) solution to each well and incubation for 3 h at 37 °C was followed to produce formazan crystals. Then, the supernatant was removed and the products were lysed with 200 μL of DMSO. The absorbance value was recorded

at 590 nm using a microplate reader. The absorbance of the untreated cells was used as a control and its absorbance was as the reference value for calculating 100% cellular viability.

In vivo NIR-II fluorescence imaging of tumors. For tumor imaging, animals were mounted on the imaging stage in the prone position beneath the laser. NIR-II fluorescence images were collected using a liquid-nitrogen-cooled, two-dimensional InGaAs array (Princeton Instruments) for collecting photons in NIR-II. The excitation light was provided by an 808-nm diode laser (RMPC) coupled to a filtered by a 1000-nm short-pass filter. spectrum of the Q1, Q4, Q4NPs and SCH1100 solution was taken using a home-built NIR-II spectroscopy setup.

***Ex vivo* biodistribution analysis.** 96 h after injection of **Q4NPs** or 48 h after injection of **SCH1100**, U87MG xenograft mice or PC-3 mice (n = 3 per group) were sacrificed, the major organs were collected. The NIR-II fluorescent signal of each organ was then collected.

Chemical synthesis and characterization

Synthesis of Q1 and Q2

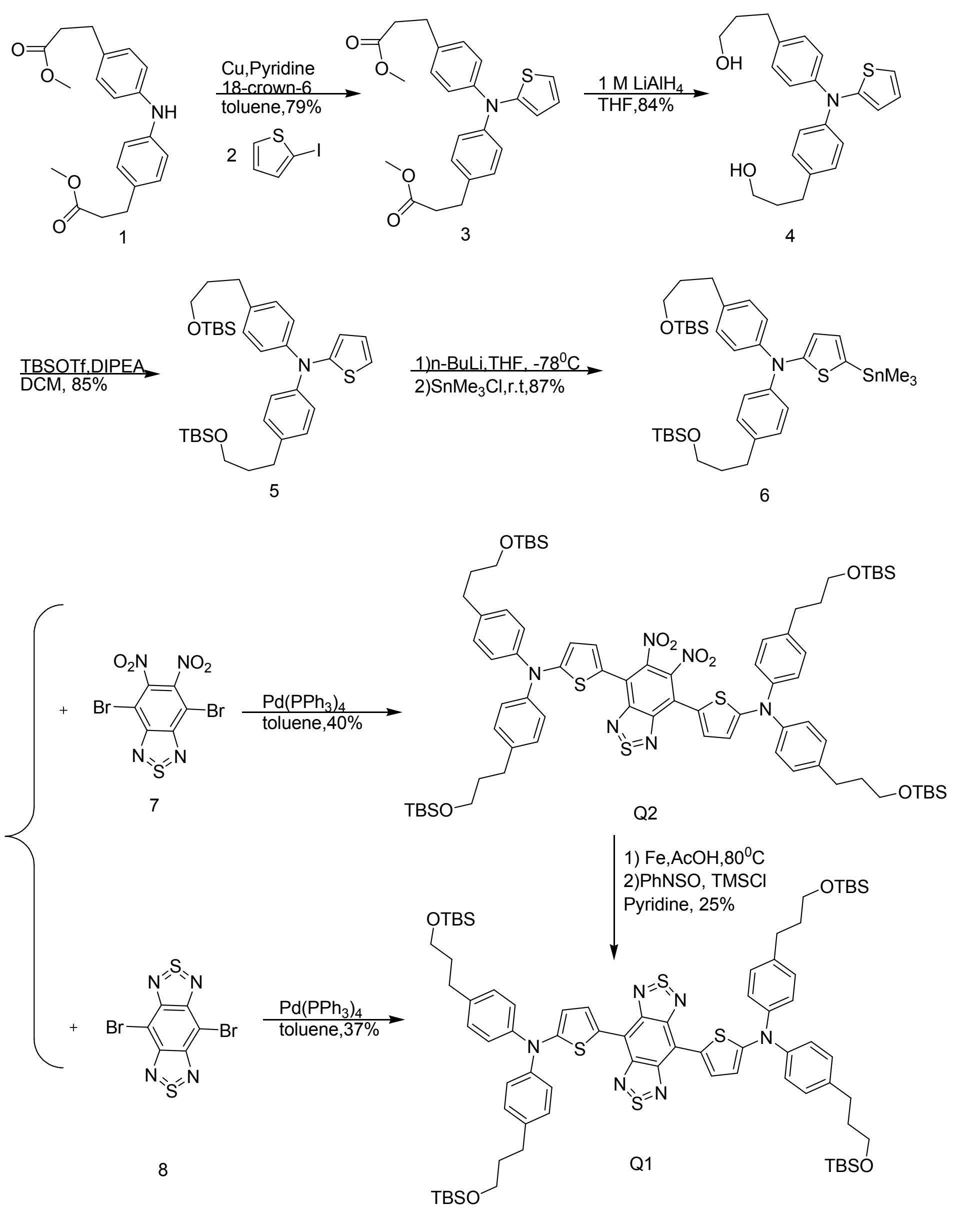

Synthesis of compound 3: A solution of compound **1** (450 mg, 1.32 mmol) in dry toluene (30 mL) was bubbled with argon for 10 min. 2-Iodothiophene (553 mg, 2.64 mmol), 18-crown-6 (34 mg, 0.132 mmol), copper (220 mg, 0.264 mmol) and pyridine (208 mg, 0.264 mmol) were added subsequently and stirred at 100°C for 18h. The reaction was monitored by TLC plate and the resulting slurry was filtered through a pad of silica gel after the complete conversion of the starting material. The resulting filtrate was evaporated *in vacuo* and was subjected to column chromatography (silica gel, petroleum: EtOAc = 50 : 1) to afford 442 mg of compound **3** as a yellow solid, Yield 79 %. ^{1}H NMR (400 MHz, $CDCl_3$) δ 7.12 – 6.99 (m, 8H), 6.97 (dd, J = 5.6, 1.2 Hz, 1H), 6.86 (dd, J = 5.6, 3.7 Hz, 1H), 6.68 (dd, J = 3.7, 1.3 Hz, 1H), 3.68 (s, 6H), 2.90 (t, J = 7.9 Hz, 4H), 2.62 (t, J = 7.9 Hz, 4H); ^{13}C NMR (101 MHz, $CDCl_3$) δ 173.4, 151.6, 146.3, 134.8,

129.0, 125.9, 122.3, 121.1, 120.5, 51.7, 35.8, 30.3. HRMS (ESI) Calcd for: $C_{24}H_{26}NO_4S^+$ ($[M+H]^+$): 424.1582, found: 424.1567.

Synthesis of compound 4: To a solution of compound **3** (120 g, 0.28 mmol) in anhydrous tetrahydrofurane (10 mL) at 0 °C was added lithium aluminium hydride (1M, 0.56 mL, 0.56 mmol) dropwise and maintained the reaction at 0 °C for 1 h. Then the suspension was warmed to 40 °C for 2 h. The reaction mixture was cooled to ambient temperature and water was added dropwise to the reaction. The resulting mixture was filtered and extracted with diethyl ether (7 mL ×3) 3 times. The combined organic layers were washed with water and sat. brine, dried over Na_2SO_4, concentrated under vacuum to give a colorless oil (87.6 mg, 84 %) for next step reaction without purification. ^{1}H NMR (400 MHz, $CDCl_3$) δ 7.05 (dd, *J* = 19.8, 8.5 Hz, 8H), 6.95 (d, *J* = 5.5 Hz, 1H), 6.86 (dd, *J* = 5.6, 3.7 Hz, 1H), 6.67 (d, *J* = 3.6 Hz, 1H), 3.68 (t, *J* = 6.4 Hz, 4H), 2.72 – 2.56 (m, 4H), 1.88 (dt, *J* = 13.5, 6.6 Hz, 4H). ^{13}C NMR (101 MHz, $CDCl_3$) δ 151.9, 146.1, 136.2, 129.1, 125.9, 122.3, 120.7, 120.2, 62.3, 34.2, 31.4. HRMS (ESI) Calcd for: $C_{22}H_{26}NO_2S^+$ ($[M+H]^+$): 368.1684, found: 368.1681.

Synthesis of compound 5: *t*-Butylmethylsilyl triflate (250 mg, 0.944 mmol) was added to a solution of compound **4** (87 mg, 0.236 mmol) and ethyldiisopropylamine (122 mg, 0.944 mmol) in 6 mL of CH_2Cl_2 at 0 ℃. After 2.5 h, the reaction was quenched with saturated $NaHCO_3$ (20 mL) and diluted with CH_2Cl_2 (10 mL). The mixture was extracted with CH_2Cl_2 (10 mL×3). The combined organic fractions were dried over $MgSO_4$ and concentrated under reduced pressure. Purification by column chromatography (petroleum : EtOAc = 50 : 1) of the crude residue afforded the titled compound **5** (120 mg) as a light yellow oil in 85% yield. ^{1}H NMR (400 MHz, $CDCl_3$) δ 7.12 – 6.99 (m, 8H), 6.94 (dd, *J* = 5.6, 1.3 Hz, 1H), 6.86 (dd, *J* = 5.6, 3.7 Hz, 1H), 6.66 (dd, *J* = 3.7, 1.3 Hz, 1H), 3.64 (t, *J* = 6.3 Hz, 4H), 2.67 – 2.57 (m, 4H), 1.82 (dq, *J* = 9.4, 6.4 Hz, 4H), 0.90 (d, *J* = 2.7 Hz, 18H), 0.05 (s, 12H). ^{13}C NMR (101 MHz, $CDCl_3$) δ 146.0, 136.6, 129.1, 125.8, 122.3, 120.4, 119.9, 62.4, 34.4, 31.4, 26.0, 18.4, -5.2. HRMS (ESI) Calcd for: $C_{34}H_{54}NO_2SSi_2^+$ ($[M+H]^+$): 596.3413, found: 596.3429.

Synthesis of compound 6: To a solution of compound **5** (100 mg, 0.168 mmol) in dry THF (10 mL) at -78°C was added n-butyllithium (2.4 M in THF, 0.11 mL, 0.252 mmol) dropwise. After 30 min, a solution of trimethyltin chloride (66.96 mg, 0.336 mmol) in dry THF (1 mL) was added and

the reaction mixture was warmed to ambient temperature for 12 h. After the complete conversion of the starting material (monitored by TLC analysis, Al_2O_3), the reaction was quenched with water (10 mL) and extracted with ethyl acetate (3 × 10 mL). The combined organic layers were dried over Na_2SO_4, and concentrated under reduced pressure. Compound **6** was obtained as brown oil which can be used in the next step without further purification. Yield 87 %, 110 mg. ^{1}H NMR (400 MHz, $CDCl_3$) δ 7.12 – 6.99 (m, 8H), 6.94 (dd, *J* = 5.6, 1.3 Hz, 1H), 6.86 (dd, *J* = 5.6, 3.7 Hz, 1H), 6.66 (dd, *J* = 3.7, 1.3 Hz, 1H), 3.64 (t, *J* = 6.3 Hz, 4H), 2.67 – 2.57 (m, 4H), 1.82 (dq, *J* = 9.4, 6.4 Hz, 4H), 0.90 (d, *J* = 2.7 Hz, 18H), 0.05 (s, 12H).

General Procedure for synthesis of Q1 and Q2

A mixture of compound **6** (2.5 equiv.) and compounds **7** or **8** (1 equiv.) in toluene was bubbled with argon for 20 min. $Pd(PPh_3)_4$ (0.1 equiv.) was added to the above mixture. The mixture was heated to 80℃ and maintained at 80℃ for additional 13 h. The reaction mixture was cooled to ambient temperature and the solvent was removed in vacuo. The resulting residue was dissolved in CH_2Cl_2 and filtered through a pad of silica gel. The filtrate was evaporated in vacuo and subjected to column chromatography to afford the titled product.

Synthesis of Q1: silica gel column; MeOH/ CH_2Cl_2=1:1, yield 37%.

^{1}H-NMR (400 MHz, $CDCl_3$) δ 7.44 – 7.37 (m, 1H), 7.18 – 7.13 (m, 1H), 6.99 (dd, *J* = 19.6, 9.4 Hz, 16H), 6.84 – 6.78 (m, 1H), 6.61 (d, *J* = 3.4 Hz, 1H), 3.59 (t, *J* = 6.3 Hz, 8H), 2.65 – 2.48 (m, 8H), 1.78 (dd, *J* = 15.2, 7.1 Hz, 8H), 0.85 (s, 36H), -0.00 (d, *J* = 2.8 Hz, 18H). MALDI-TOF-MS Calcd for: $C_{74}H_{105}N_6O_4S_4Si_4^+$ ($[M+H]^+$): 1381.6157, found: 1382.2292.

^{13}C NMR (101 MHz, $CDCl_3$) δ 153.7, 143.6, 138.3, 131.0, 128.6, 128.2, 127.5, 124.0, 123.0, 61.3, 33.2, 28.7, 25.0, 17.3, -6.7.

Synthesis of Q2: silica gel column; petrolum/EtOAc=20:1, yield 40%. ^{1}H NMR (400 MHz, $CDCl_3$) δ 7.33 (d, *J* = 4.4 Hz, 2H), 7.25 – 7.10 (m, 16H), 6.43 (dd, *J* = 7.6, 4.4 Hz, 2H), 3.65 (t, *J* = 6.3 Hz, 8H), 2.72 – 2.63 (m, 8H), 1.84 (dq, *J* = 9.5, 6.3 Hz, 8H), 0.91 (s, 36H), 0.06 (s, 24H). ^{13}C NMR (101 MHz, $CDCl_3$) δ 162.9, 152.3, 150.9, 144.1, 140.0, 132.7, 129.7, 125.0, 124.6, 122.7, 116.2, 113.6, 103.5, 62.3, 34.2, 31.6, 29.7, 26.0, 18.4, 1.1 , -5.2. HRMS (ESI) Calcd for: $C_{74}H_{105}N_6O_8S_3Si_4^+$ ($[M+H]^+$): 1413.6233, found: 1413.6289.

Synthesis of Q3

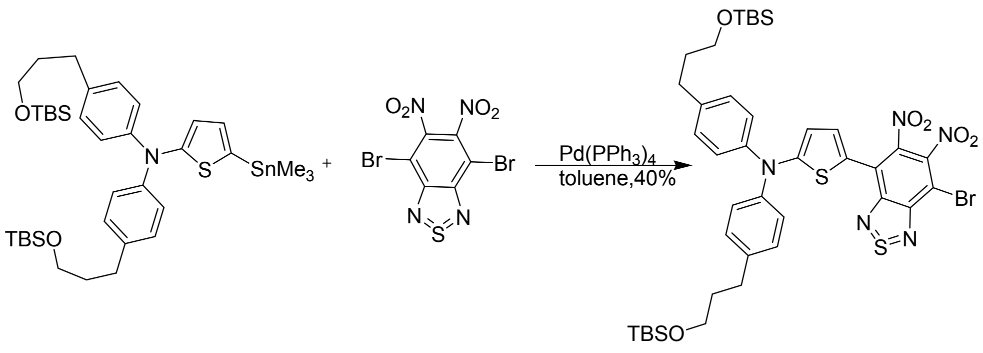

Synthesis of compound 9: A mixture of compound **6** (1.5 equiv.) and compounds **7** (1 equiv.) in toluene was bubbled with argon for 20 min. $Pd(PPh_3)_4$ (0.1 equiv.) was added to the above mixture. The mixture was heated at 80℃ for 13 h, and the solvent was removed in vacuo. The residue was dissolved in CH_2Cl_2, and the resulting solution was filtered through a pad of silica gel. The filtrate was evaporated in vacuo and subjected to column chromatography to afford the titled product. Silica gel column; petrolum/EtOAc=20:1, a blue solid, yield 33%. ^{1}H NMR (400 MHz, $CDCl_3$) δ 7.30 – 7.17 (m, 8H), 7.13 – 6.96 (m, 1H), 6.47 (d, J = 4.3 Hz, 1H), 3.69 (t, J = 6.2 Hz, 4H), 2.76 – 2.63 (m, 4H), 1.93 – 1.82 (m, 4H), 0.94 (s, 18H), 0.09 (s, 12H). ^{13}C NMR (101 MHz, $CDCl_3$) δ 160.6, 151.9, 144.6, 140.3, 139.2, 130.8, 129.6, 124.6, 118.5, 118.0, 114.4, 62.3, 34.3, 31.5, 26.0, 18.4, -5.2. HRMS (ESI) Calcd for: $C_{40}H_{53}BrN_5O_6S_2Si_2^+$ ($[M+H]^+$): 898.2159, found: 898.2174.

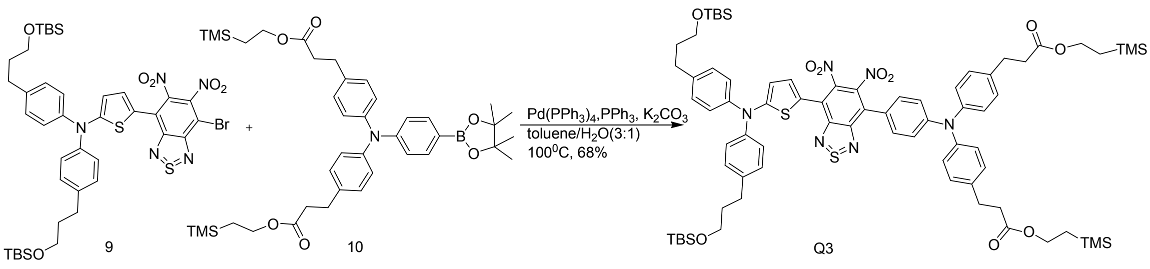

Synthesis of Q3: To a solution of compound **9** (20 mg, 0.023 mmol) and compound **10** (20 mg, 0.027 mmol) in toluene (3 mL) was added $Pd(PPh_3)_4$ (2.6 mg, 0.0023 mmol) and triphenylphosphine (1.2 mg, 0.0046 mmol) under an argon atmosphere in 25 mL sealed tube. Potassium carbonate (6.3 mg, 0.046 mmol) in 1 mL distilled H_2O was added to the reaction solution. The mixture was stirred for 18 h at 80 °C. After cooling to room temperature, the mixture was poured into water and extracted with EtOAc (3 x 6 mL). The organic layer was washed with saturated aqueous brine before being dried over $MgSO_4$. After evaporation of the

solvent, the residue was purified by column chromatography on silica gel with ethyl acetate/petroleum ether (1:15) as the eluent to afford the product Q3 (22 mg, 68 %) as a purple solid. ^{1}H NMR (400 MHz, $CDCl_3$) δ 7.33 (d, J = 4.1 Hz, 1H), 7.24 (d, J = 4.2 Hz, 1H), 7.17 (d, J = 11.2 Hz, 4H), 7.13 – 7.07 (m, 10H), 6.99 (d, J = 8.4 Hz, 4H), 6.50 (d, J = 4.2 Hz, 1H), 6.44 (d, J = 4.2 Hz, 1H), 4.27 – 4.16 (m, 4H), 3.66 (d, J = 8.9 Hz, 4H), 2.92 (t, J = 7.9 Hz, 4H), 2.68 – 2.60 (m, 8H), 1.86 – 1.80 (m, 4H), 1.05 – 0.97 (m, 4H), 0.92 (s, 18H), 0.07 (d, J = 5.2 Hz, 30H). ^{13}C NMR (101 MHz, CDCl3) δ 160.6, 151.9, 144.6, 140.3, 139.2, 130.8, 129.6, 124.6, 118.5, 117.9, 114.4, 62.3, 34.3, 31.6, 26.0, 18.4, -5.2. HRMS (ESI) Calcd for: $C_{74}H_{99}BrN_6O_{10}S_2Si_4^+$ ($[M+H]^+$): 1407.5941, found: 1407.5931.

Synthesis of Q4

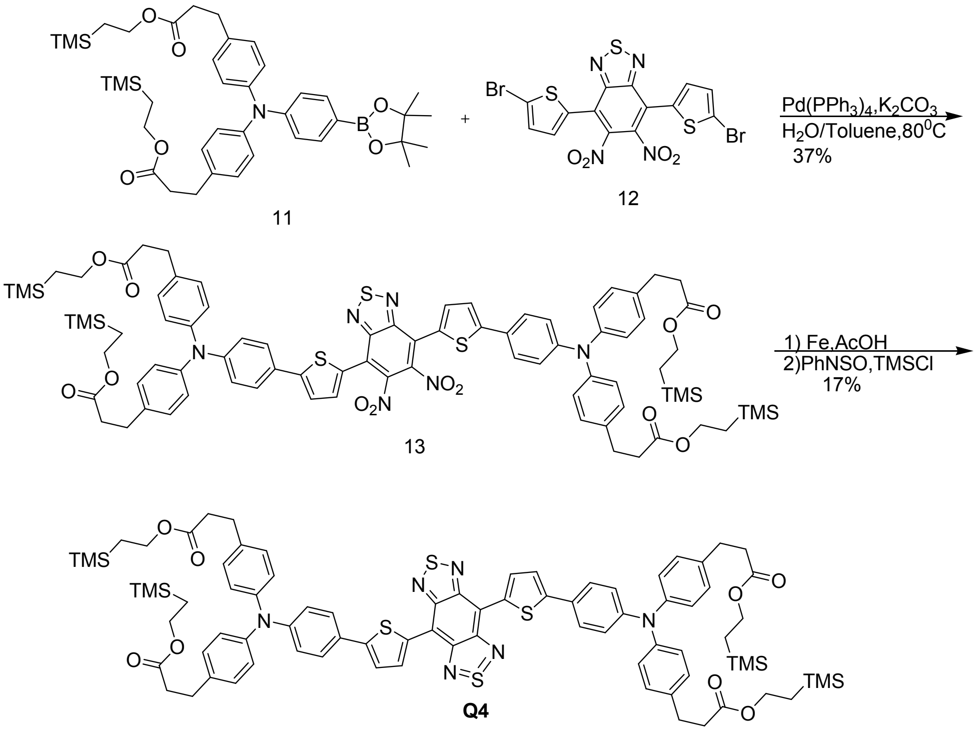

Synthesis of compound 13: To a solution of compound **11** (40 mg, 0.055 mmol) and compound **12** (62 mg, 0.11 mmol) in toluene (6 mL) was added $Pd(PPh_3)_4$ (6.5 mg, 0.0056 mmol) under an argon atmosphere in 25 mL sealed tube. Potassium carbonate (20 mg, 0.14 mmol) in distilled H_2O (2 mL) was added to reaction solution. The mixture was stirred for 18 h at 80 °C. After cooling to room temperature, the mixture was poured into water and extracted with EtOAc (3 x 12 mL). The organic layers were washed with saturated aqueous brine before being dried over $MgSO_4$. After evaporation of the solvents, the residue was purified by column chromatography on silica gel with

ethyl acetate/petroleum ether (1:4) as the eluent to afford product **3** (31 mg, 37 %) as a blue solid. ^{1}H-NMR (400 MHz, $CDCl_3$) δ 7.50 (d, *J* = 8.2 Hz, 6H), 7.29 (d, *J* = 3.8 Hz, 2H), 7.12 (d, *J* = 8.2 Hz, 8H), 7.07 – 7.00 (m, 12H), 4.23 – 4.14 (m, 8H), 2.92 (t, *J* = 7.7 Hz, 8H), 2.61 (t, *J* = 7.8 Hz, 8H), 1.02 – 0.94 (m, 8H), 0.05 (s, 36H). ^{13}C -NMR (101 MHz, CDCl3) δ 174.5, 153.5, 152.6, 150.0, 146.7, 137.4, 133.6, 130.8, 129.1, 128.4, 127.6, 126.5, 124.4, 123.7, 121.8, 64.2, 54.9, 37.5, 31.8, 18.8, 0.0. HRMS (ESI) Calcd for: $C_{82}H_{97}N_6O_{12}S_3Si_4^+$ ($[M+H]^+$): 1565.5403, found: 1565.5492.

Synthesis of Q4: 10 mL round flask was added compound **3** (15 mg, 0.0096 mmol), iron powder (32 mg, 0.57 mmol), AcOH (2.5 mL). The reaction mixture was heated to 100 °C for 4 h and then cooled to room temperature. The reaction solution was changed from blue to yellow. The reaction was neutralized with sat. $NaHCO_3$ and extracted with EtOAc (3 × 7 mL). The combined organic layers were washed with water (10 mL), dried over anhydrous $MgSO_4$ and evaporated in vacuum. The resulting brown oil was used for the next step without further purification. To a brownish solution in anhydrous pyridine (2 mL) was added N-thionylaniline (3 mg, 0.022 mmol) and TMSCl (10.5 mg, 0.428 mol).The solution was heated in an oil bath at 80℃ for 12 h. The reaction was allowed to cool, poured into iced water, extracted with EtOAc (3 x 5 mL). The combined organic layers were washed with water (10 mL), dried over anhydrous $MgSO_4$ and evaporated in vacuum. The residue was purified by column chromatography on silica gel with petroleum ether/EtOAc (3:1) to yield the product **Q4** as a blue solid (2.3 mg, 17 % yield). ^{1}H NMR (400 MHz, $CDCl_3$) δ 7.76 (d, *J* = 3.8 Hz, 2H), 7.52 (d, *J* = 8.6 Hz, 4H), 7.31 (d, *J* = 3.8 Hz, 2H), 7.07 (dd, *J* = 25.4, 8.3 Hz, 20H), 4.27 – 4.10 (m, 8H), 2.92 (t, *J* = 7.9 Hz, 8H), 2.61 (t, *J* = 7.8 Hz, 8H), 1.06 – 0.93 (m, 8H), 0.05 (s, 36H). MALDI-TOF-MS Calcd for: $C_{82}H_{97}N_6O_8S_4Si_4^+$ ($[M+H]^+$): 1533.5250, found: 1533.5594.

Synthesis of Q4NPs

The Q4 was dissolved in THF at a concentration of 100 µg/mL and mixed with an aqueous solution of DSPE-mPEG(5 kDa) at a concentration of 1 mg/mL with 1 : 9 volume ratio was stirred at room temperature for 4 h. Then, the mixture was dialysed against water to remove THF and make a THF-free, clear aqueous solution of **Q4NPs**. To remove aggregates formed during dialysis,

the suspension was ultracentrifuged for 30 min at 300,000 g and only the supernatant was retained. Free unbound surfactant in the solution was removed through 30 kDa centrifugal filters (Amicon) without cause any instability to the **Q4NPs.** The amount of Q4 in Q4NPs was calculated based on the UV-Vis measurement of Q4.

Synthesis of RM26

Peptide RM26 (D-f-Q-W-A-V-G-H-Sta-L-NH_2) was synthesized on Tentagel S RAM resin using traditional Fmoc solid-phase peptide chemistry. After deprotection and cleavage from the resin using 93% TFA, 5% Tips, and 2% H_2O for 2 h, the peptide was precipitated in cold Et_2O and washed with Et_2O three times. The dried peptide was purified by prep-HPLC. MS Calcd for: Calcd for RM1: $C_{55}H_{81}N_{14}O_{11}^+$ ($[M+H]^+$): 1113.6, found: m/z 1113.7,

Synthesis of Q4-1:

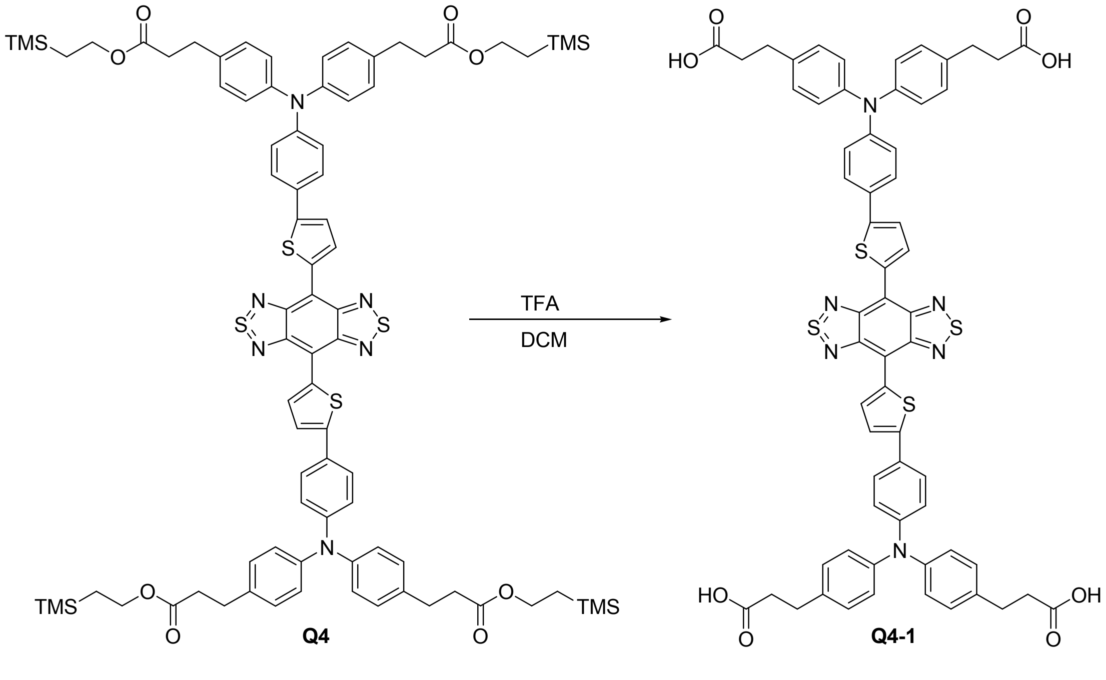

To a solution of compound Q4 (1 mg) in DCM (0.1 mL) was added TFA (0.1 mL) at 0°C. The reaction mixture was slowly warmed to ambient temperature. The reaction was completed in 30 min by TLC analysis. The solvent was removed in vacuo and the crude product was washed by acetonitrile to yield the crude product as a green semi-solid. The crude product was purified by Prep-HPLC to obtain the desired product Q4-1 (0.3 mg, 40%). ESI-MS Calcd for: $C_{62}H_{49}N_6O_8S_4^+$ ($[M+H]^+$): 1132.2, found: 1133.1.

Synthesis of **SCH1100**

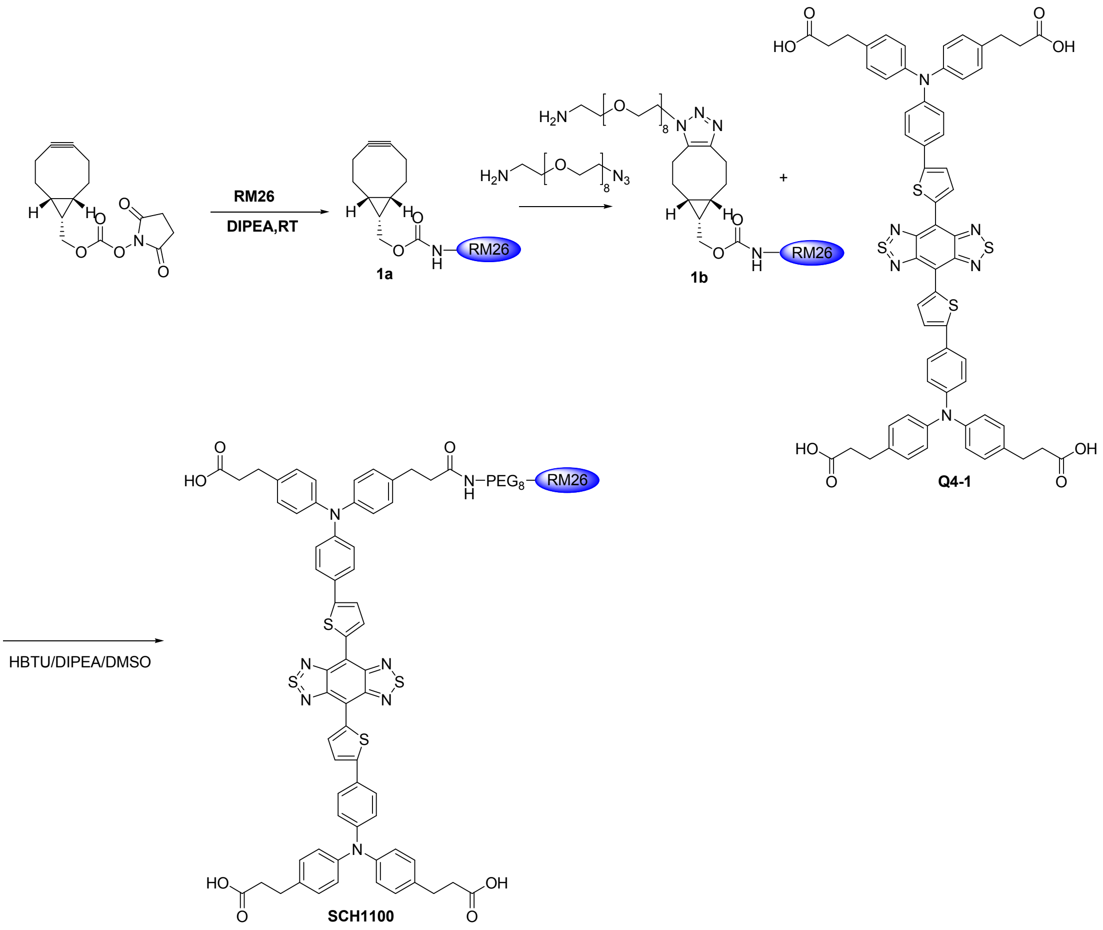

Synthesis of 1a: To a solution of BCN-NHS (1.5 mg, 0.005 mmol) and RM26 peptide (5.56 mg, 0.005 mmol, 1.0 equiv) in DMF (c=0.3 M) at room temperature was added DIPEA (2 equiv). The reaction mixture was stirred at this temperature for 12 h. The crude product was purified by HPLC. Lyophilization of the purified material gave 3.22 mg (50 %) of **1a** as a white semi-solid. MS Calcd for: $C_{66}H_{94}N_{14}O_{13}^{+}$ ($[M+H]^{+}$): 1290.7, found: ESI-MS: m/z 1290.3.

Synthesis of 1b: To a solution of compound **1a** (2.58 mg, 0.002 mmol) and PEG8-azide (1.75 mg, 0.004 mmol, 1.0 equiv) were in DMF at room temperature. The reaction mixture was stirred at this temperature for 12 h. The crude product was purified by HPLC. Lyophilization of the purified material gave 2.07 mg (60 %) of **1b** as white Powder. MS Calcd for: $C_{84}H_{132}N_{18}O_{21}^{+}$ ($[M+H]^{+}$): 1728.9, found: ESI-MS: m/z 1728.4.

Synthesis of SCH1100: To a solution of compound **Q4-1** (1.13 mg, 0.001 mmol) and HBTU (0.38 mg, 0.001 mmol, 1.0 equiv) in DMF at room temperature. The reaction mixture was stirred at this temperature for 4 h. Then **1b** (1.73 mg, 0.001 mmol) was added in the reaction mixture and stirred overnight. The crude product was purified by HPLC. Lyophilization of the purified material gave

0.82 mg (30 %) of **SCH1100**. MALDI-TOF [Calcd. for $C_{146}H_{176}N_{24}O_{28}S_4$: 2843.3630, found: m/z 2844.1526.

NMR Spectra

^{1}H and ^{13}C NMR for 3

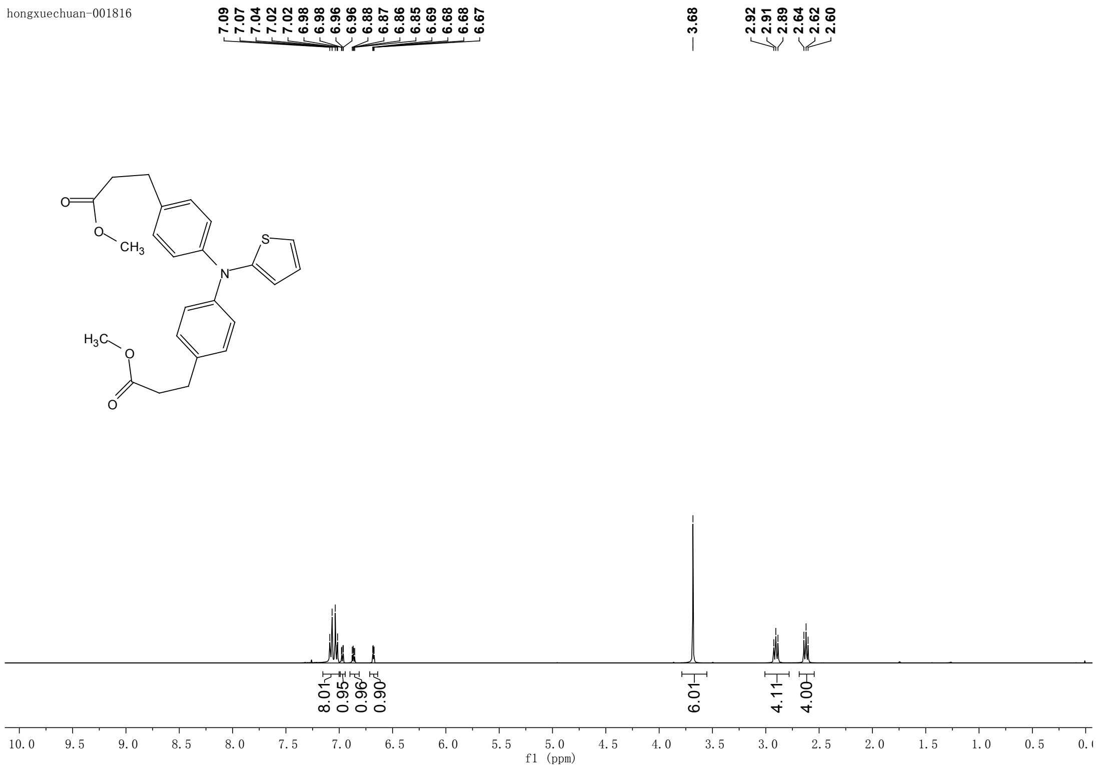

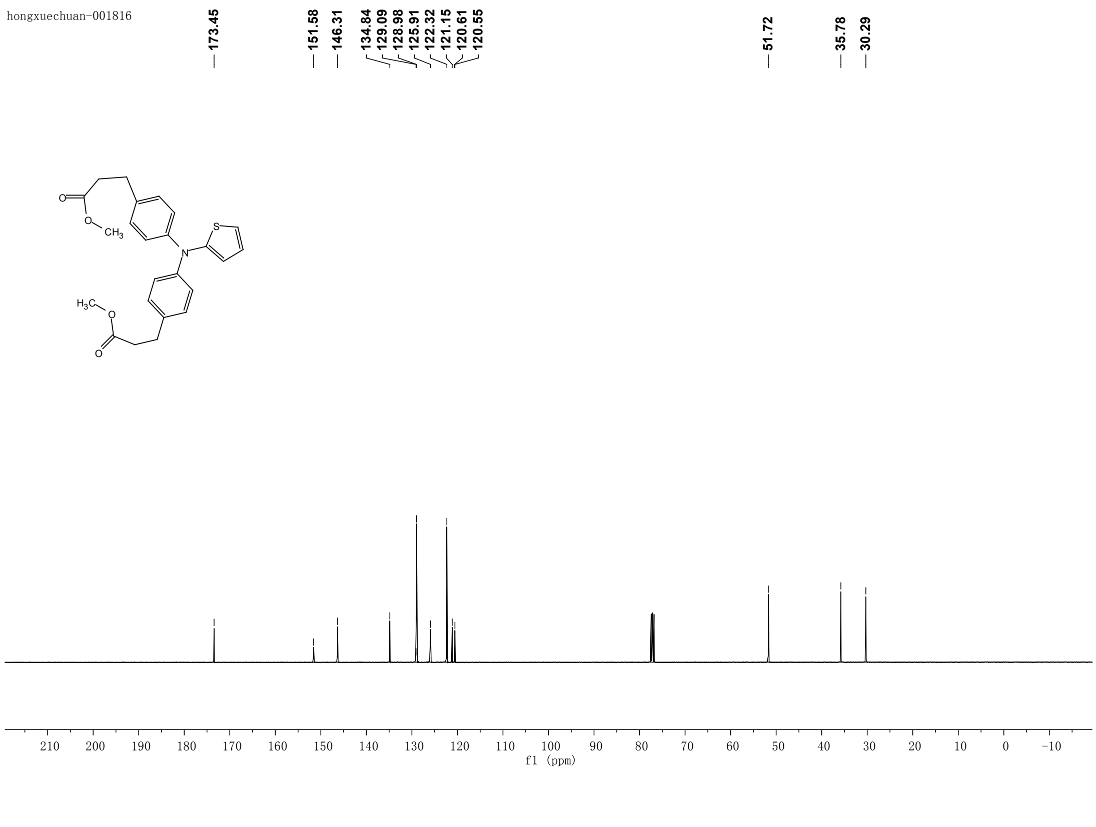

^{1}H and ^{13}C NMR for 4

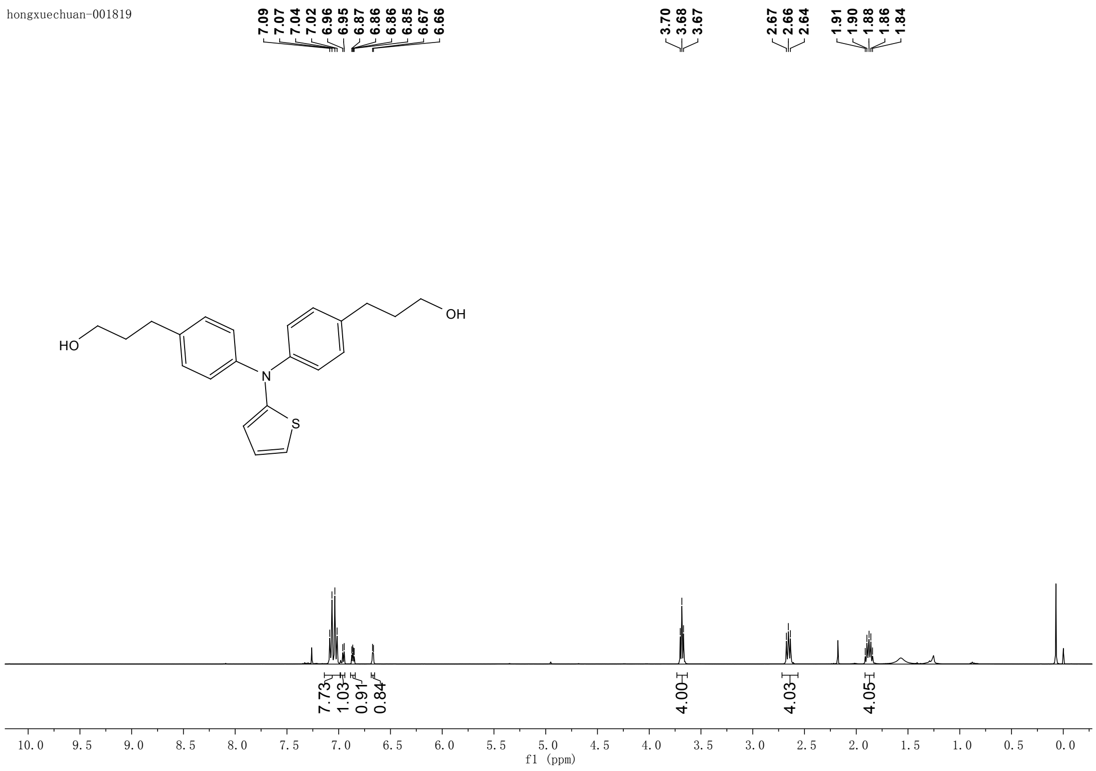

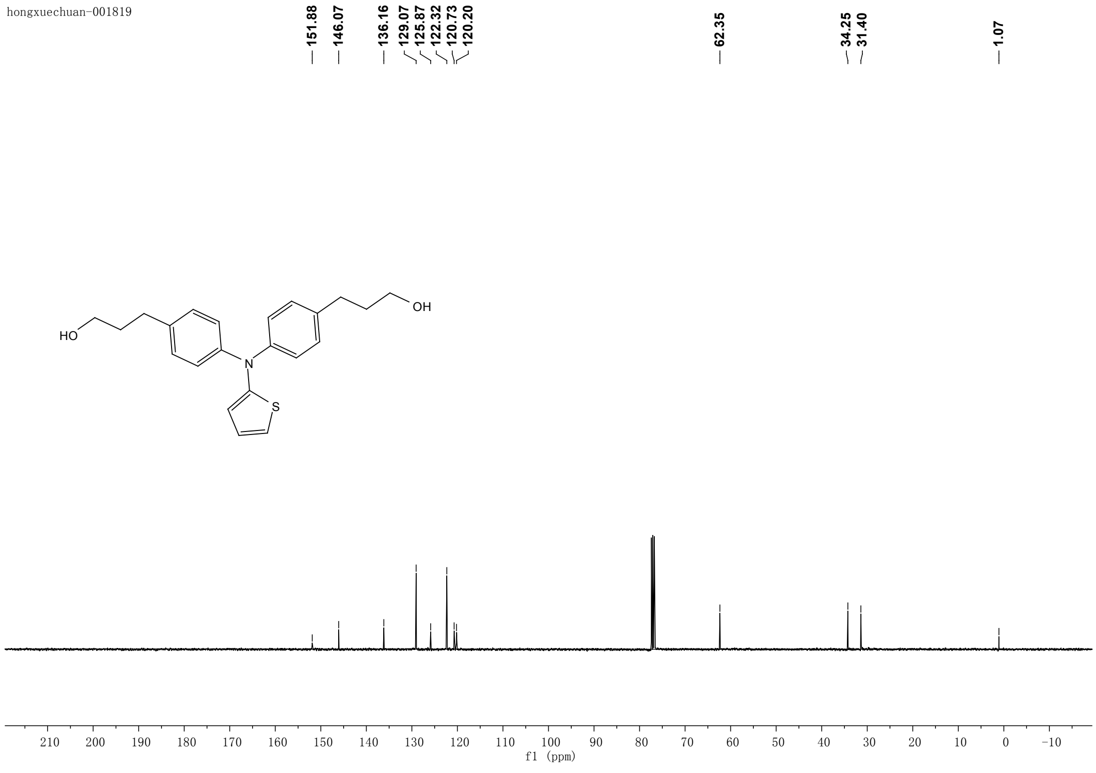

^{1}H and ^{13}C NMR for 5

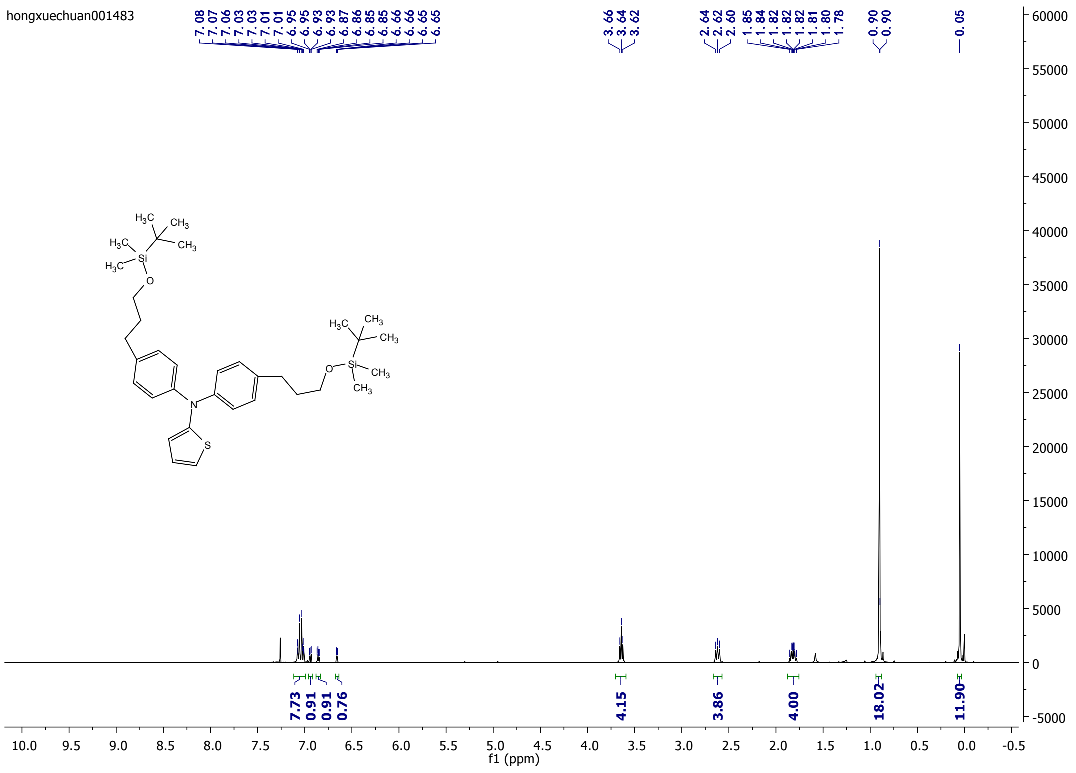

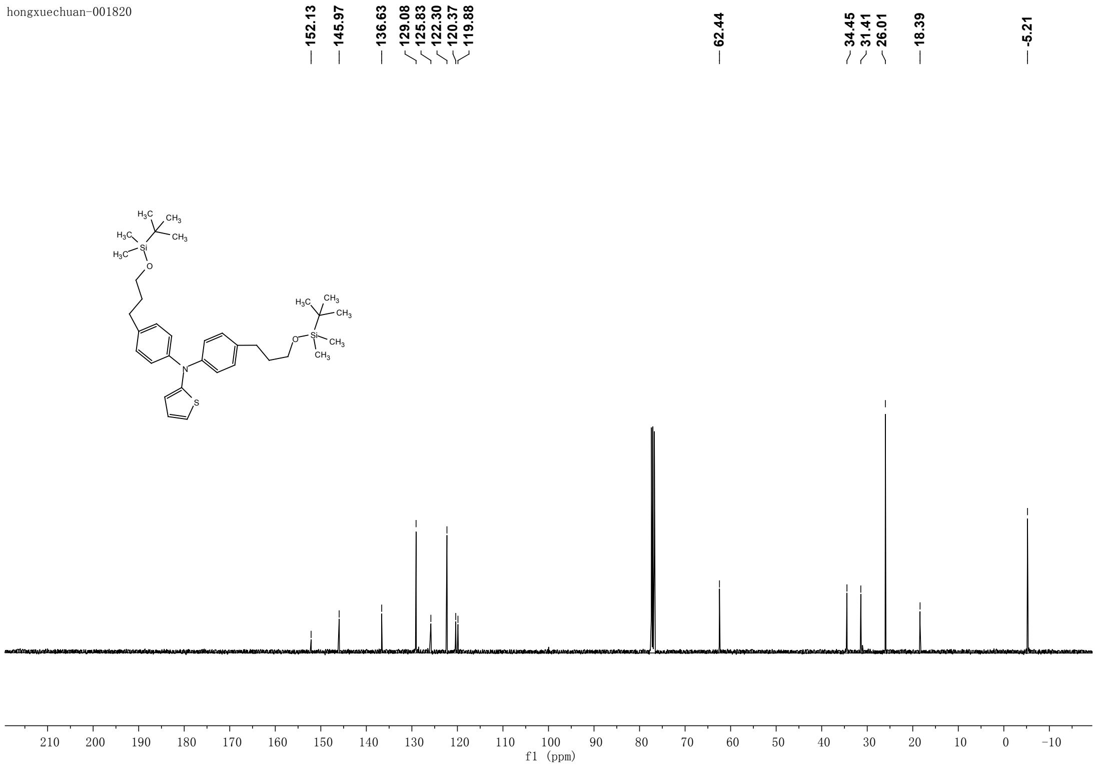

^{1}H and ^{13}C NMR for 9

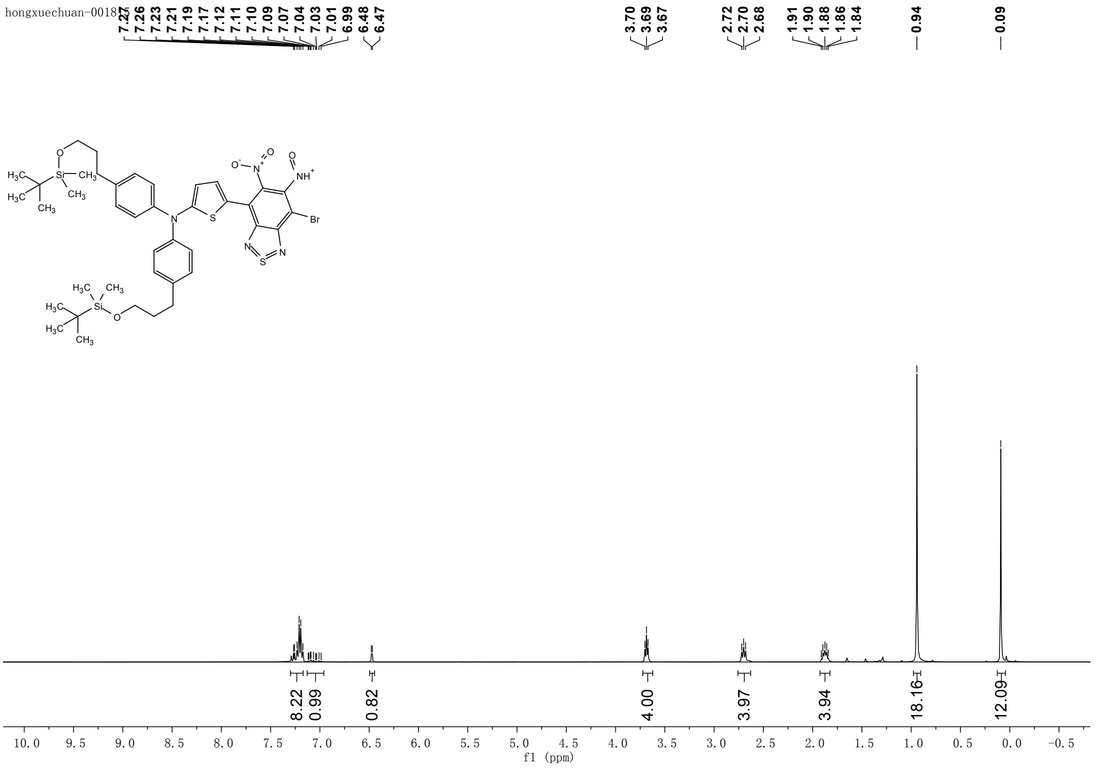

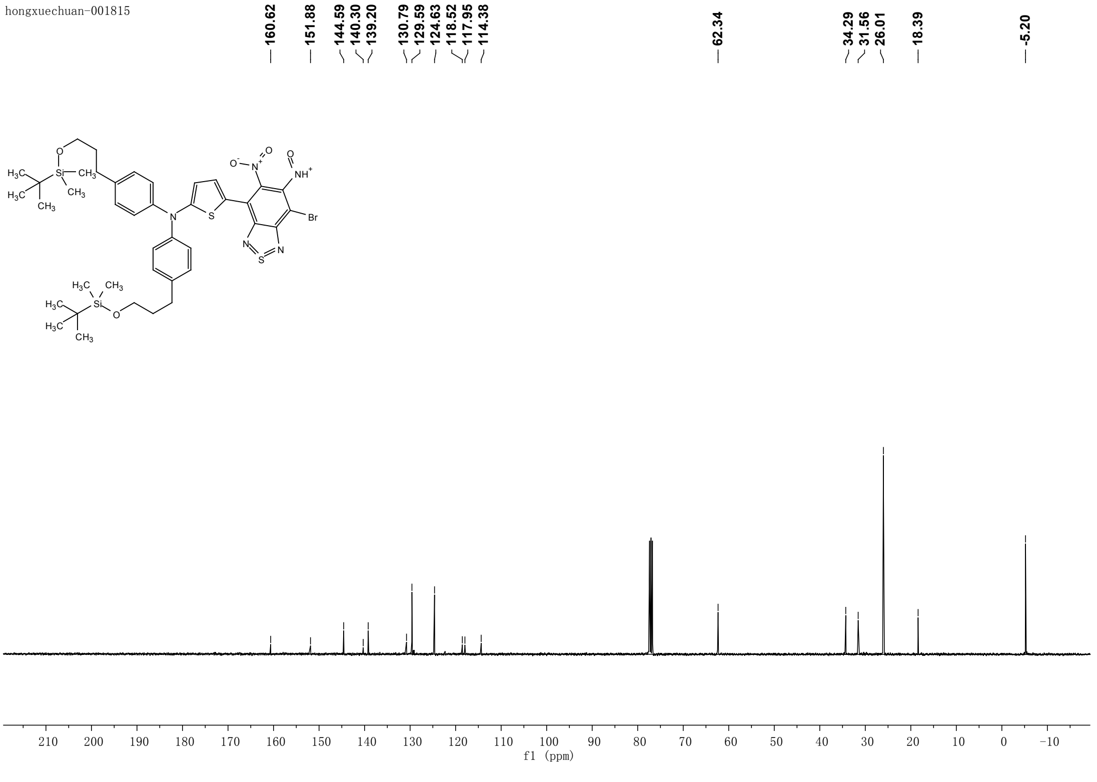

^{1}H NMR for Q1

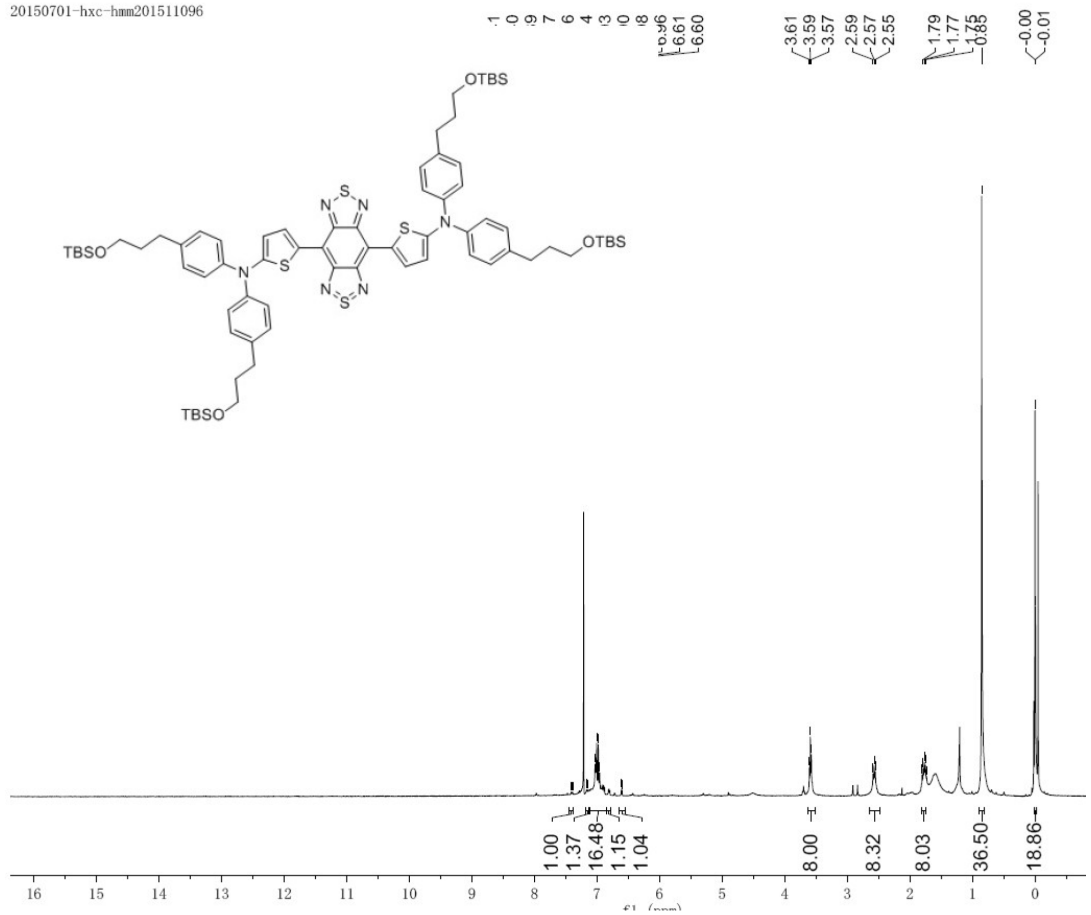
20150701-hxc-hmm201511096
6.96
6.61
6.60
3.61
3.59
3.57
2.59
2.57
2.55
1.79
1.77
1.75
0.85
-0.00
-0.01
OTBS
TBSO
1.00
1.37
16.48
1.15
1.04
8.00
8.32
8.03
36.50
18.86
16 15 14 13 12 11 10 9 8 7 6 5 4 3 2 1 0

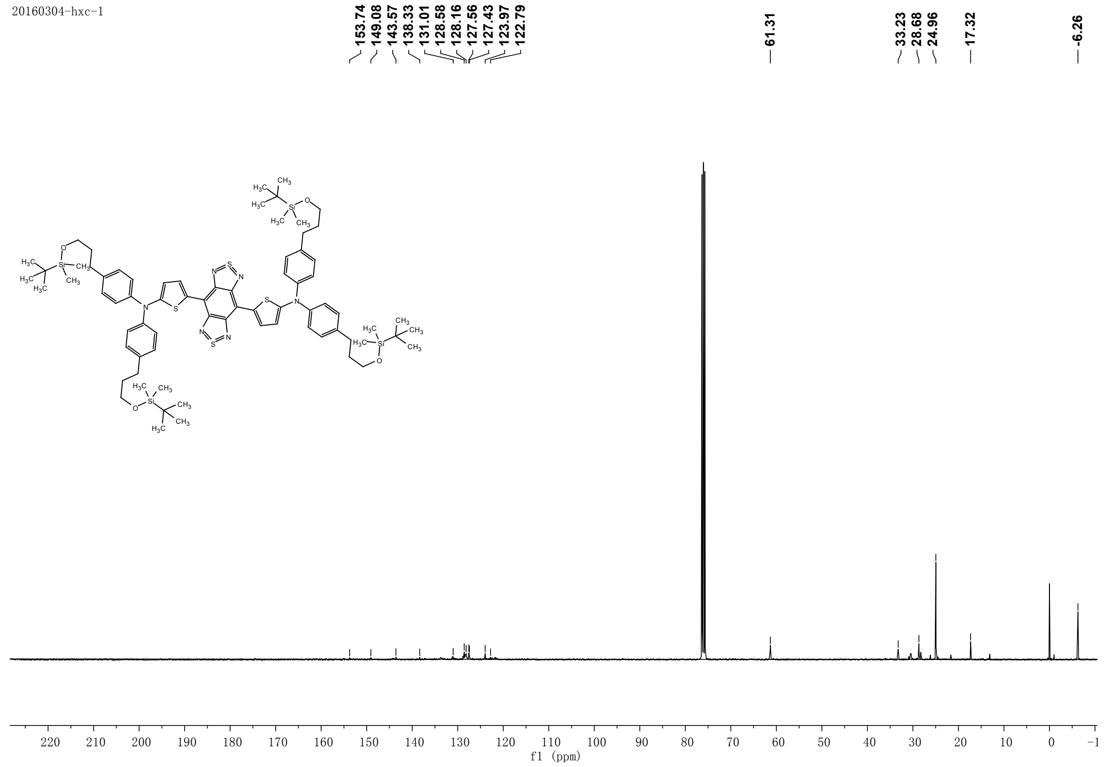
20160304-hxc-1
153.74
149.08
143.57
138.33
131.01
128.58
128.16
127.56
127.43
123.97
122.79
61.31
33.23
28.68
24.96
17.32
-6.26
220 210 200 190 180 170 160 150 140 130 120 110 100 90 80 70 60 50 40 30 20 10 0 -1
f1 (ppm)

^{1}H and ^{13}C NMR for Q2

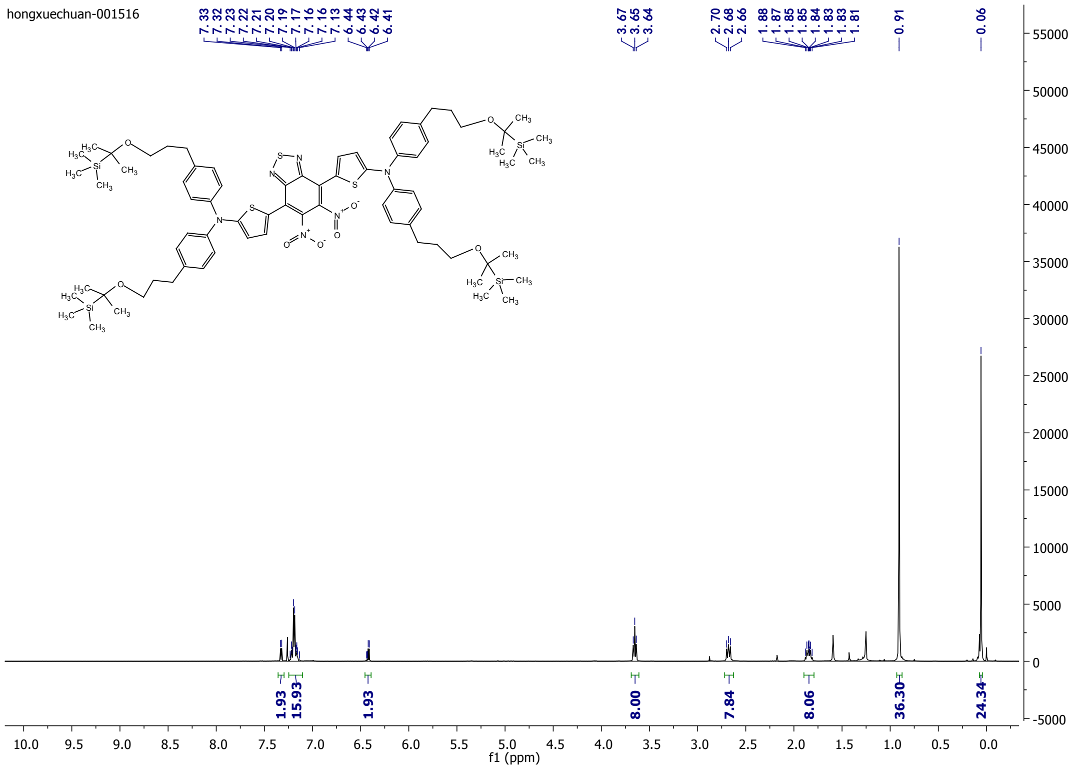

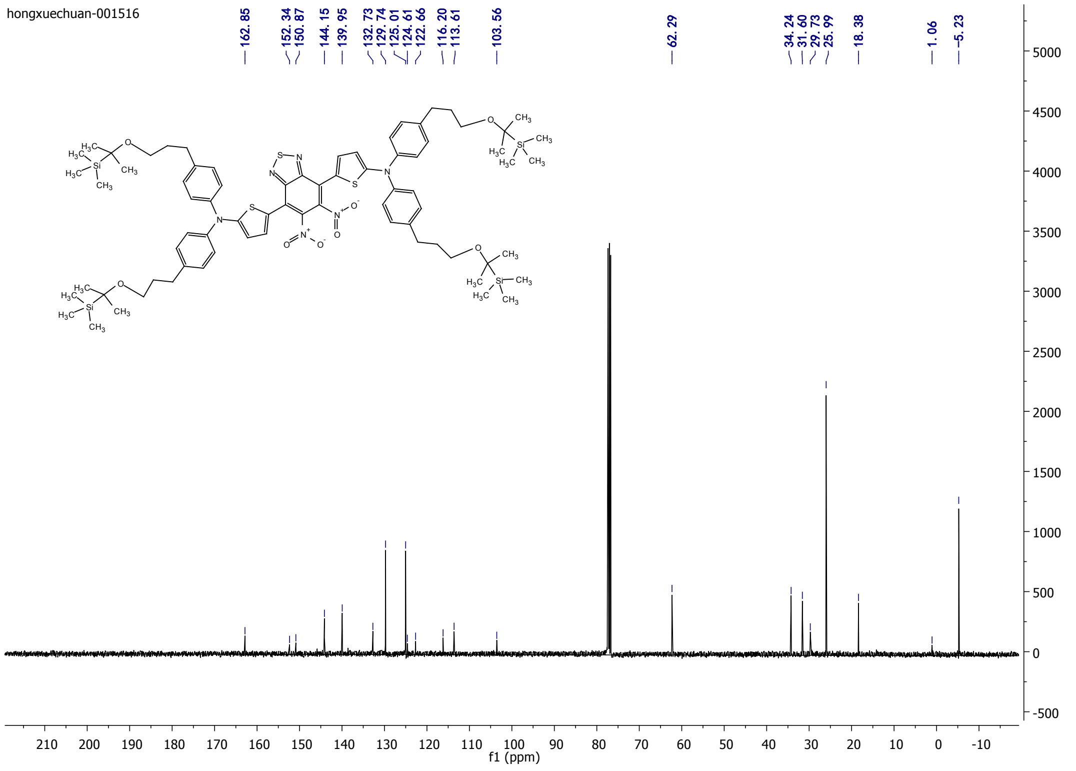

^{1}H and ^{13}C NMR for Q3

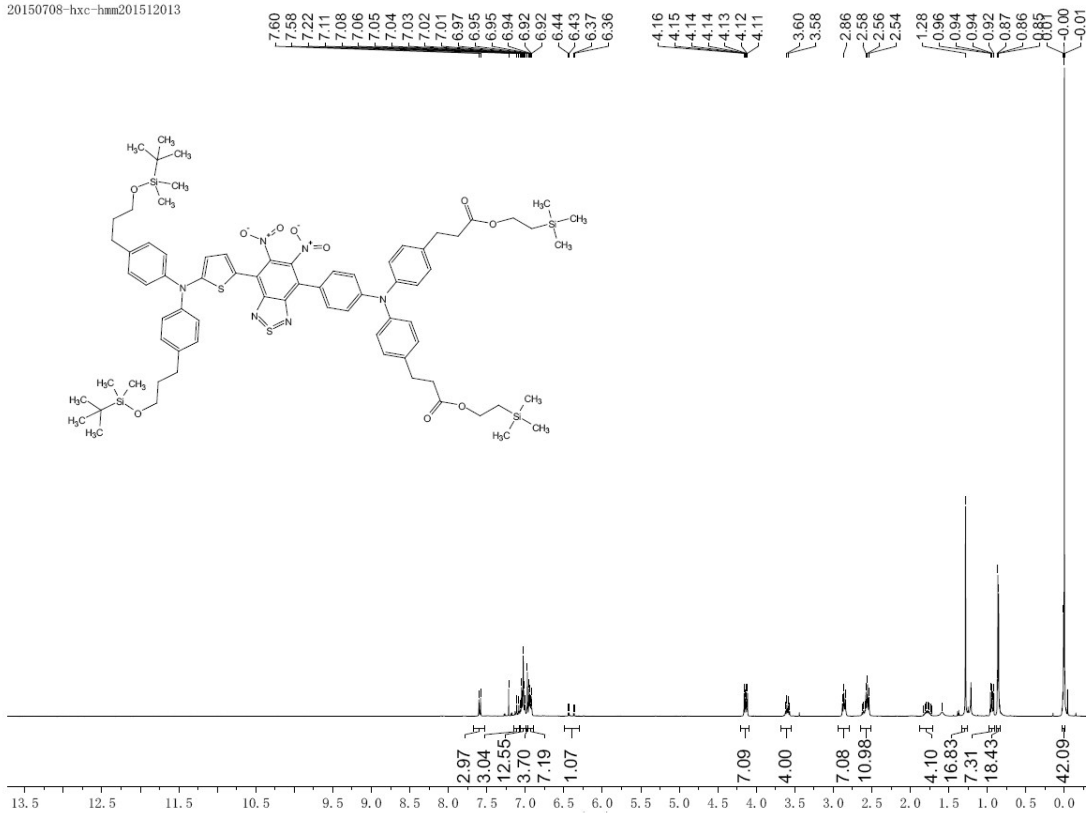

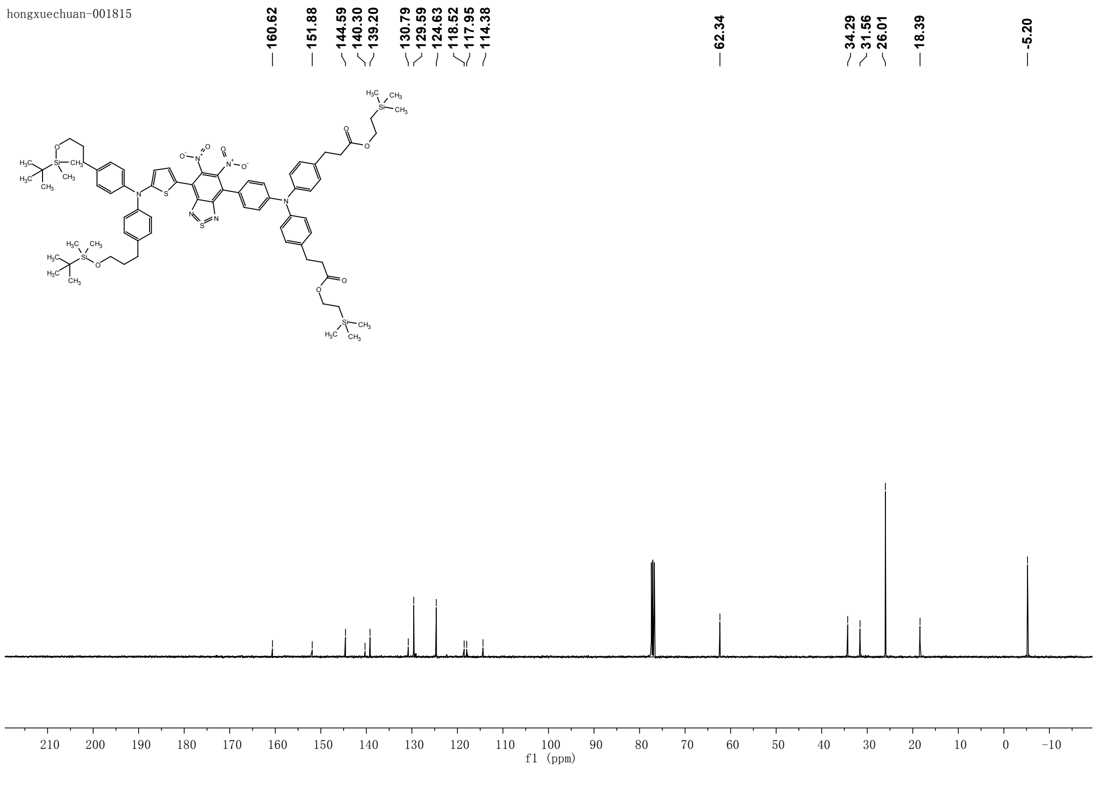

^{1}H NMR for Q4

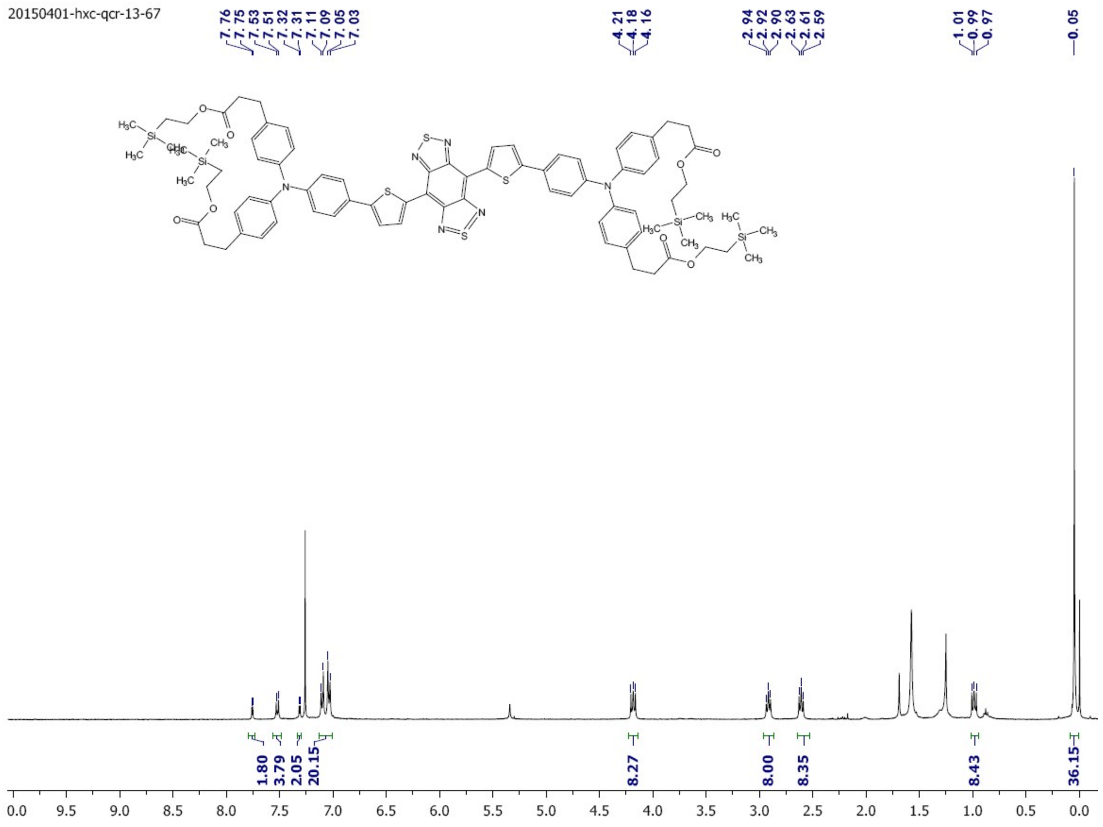

20150401-hxc-qcr-13-67
7.76
7.75
7.53
7.51
7.32
7.31
7.11
7.09
7.05
7.03
4.21
4.18
4.16
2.94
2.92
2.90
2.63
2.61
2.59
1.01
0.99
0.97
0.05
1.80
3.79
2.05
20.15
8.27
8.00
8.35
8.43
36.15
0.0 9.5 9.0 8.5 8.0 7.5 7.0 6.5 6.0 5.5 5.0 4.5 4.0 3.5 3.0 2.5 2.0 1.5 1.0 0.5 0.0
